# Multi-Omics Analysis Reveals the transforming growth factor-β Signaling-Driven Multicellular Interactions with Prognostic Relevance in Cervical Cancer Progression

**DOI:** 10.7150/jca.114505

**Published:** 2025-06-20

**Authors:** Yuhan Wang, Guangxu Cao, Huimin Zeng, Yong Zhi, Mengting Xu, Ying Wang, Min Liu, Yetian Ruan, Ka Yu Tse, Qingfeng Zhang, Jinli Gao, Zhiqiang Han, Fang Li

**Affiliations:** 1 Department of Obstetrics and Gynecology, Shanghai East Hospital, School of Medicine, Tongji University, Shanghai, China.; 2 Department of Obstetrics and Gynecology, Tongji Hospital, Tongji Medical College, Huazhong University of Science and Technology, Wuhan, Hubei, China; 3 State Key Laboratory of Cardiology and Research Center for Translational Medicine, Shanghai East Hospital, School of Medicine, Tongji University, Shanghai, China.; 4 Department of Obstetrics and Gynaecology, Queen Mary Hospital, the University of Hong Kong, Hong Kong, China.; 5 Key Laboratory of Pathogen-Host Interaction, Ministry of Education; State Key Laboratory of Cardiology and Research Center for Translational Medicine, Shanghai East Hospital; Clinical Center for Brain and Spinal Cord Research, School of Medicine, Tongji University, Shanghai, China.; 6 Department of Pathology, Shanghai East Hospital, School of Medicine, Tongji University, Shanghai, China.; 7 Department of Obstetrics and Gynecology, Shanxi Bethune Hospital, Shanxi Academy of Medical Sciences, Tongji Shanxi Hospital, Third Hospital of Shanxi Medical University, Taiyuan, Shanxi, China.

**Keywords:** cervical cancer, tumor progression, multi-omics analysis, TME, niche

## Abstract

While cervical cancer (CC) prognosis depends on tumor staging, the spatiotemporal evolution of tumor microenvironment (TME) heterogeneity during metastatic progression remains poorly characterized at single-cell resolution. We employed an integrative multi-omics approach, combining single-cell RNA sequencing (scRNA-seq; n = 11), spatial transcriptomics (ST), and bulk RNA-seq data from the TCGA-CESC cohort (n = 304), to systematically map TME remodeling across CC progression stages. scRNA-seq was performed on primary lesions from patients with localized (n = 3), regional (n = 4), and metastatic (n = 4) diseases, with in-depth analyses focusing on cellular characteristics, cell type composition alterations, functional changes, differentiation trajectories, and cell-cell interaction networks. These findings were further validated using spatial transcriptomics, bulk RNA-seq data, and multiple immunohistochemistry (mIHC) experiments. ScRNA-seq data revealed that the TME of the metastatic group displayed a distinct immunosuppressive phenotype. Three key subclusters closely linked to TME remodeling in this group were identified. Notably, a novel metastasis-associated epithelial subpopulation (Epi0_AGR2), characterized by both epithelial-mesenchymal transition (EMT) and chemokine secretory phenotypes, was discovered. Gene Set Variation Analysis (GSVA) revealed that transforming growth factor β (TGF-β) signaling activation served as its primary transcriptional driver. Additionally, a neutrophil subset with pro-tumor and immunosuppressive properties, as well as a cancer-associated fibroblasts (CAFs) subset that promoted angiogenesis, were enriched in the metastatic group. Cell-cell interaction analysis and spatial mapping further revealed the formation of coordinated Epi0-neutrophil-CAFs niches, which established TGF-β-CXCL1/2/8-OSM/OSMR feedforward loops. Importantly, a computational model derived from the TME metastatic niche signature demonstrated significant prognostic stratification in the TCGA cohort (HR = 2.5179, p = 0.0144). In all, this study provides the first comprehensive delineation of stage-specific TME dynamics in CC, revealing TGF-β-driven cellular cooperativity as a metastatic switch. The joint framework establishes a potential clinically translatable tool for precision prognosis and therapeutic targeting.

## Introduction

Cancer metastasis is associated with tumor progression and lethality in cases of cervical cancer (CC) 1. The five-year survival rates of patients with stage III and stage IV CC were only 10-40% 2. The recent introduction of immune checkpoint inhibitors (ICIs) as a treatment for advanced CC patients resulted in a significant improvement in their progression-free survival and overall survival 3-6. The response to ICIs has been shown to be closely associated with the initial tumor microenvironment (TME) 7, 8. Therefore, a comprehensive understanding of the TME in advanced CC, and the exploration of the underlying mechanisms that promote cancer progression and immunosuppression, will offer new insights on the treatment and management of CC.

Metastasis is not a simple linear cascade of events but involves multiple parallel and intersecting pathways 9-11. The phenotypes of metastasis-initiating cells (MICs) depend on the interaction among various cell types 12-14, as well as modulations of multiple signaling pathways during distinct metastatic phases, including local invasion, dissemination, and colonization 15. Among multiple signaling pathways involved in the process, transforming growth factor β (TGF-β) signaling has a crucial role in metastasis and cancer progression. It can not only stimulate epithelial mesenchymal transition (EMT) and dissemination of tumor cells 15, 16, but also create an immunosuppressive TME via inhibiting the expansion and function of immune cells 17. Taniguchi *et al.* reported the presence of an invasion niche in a squamous cell carcinoma mouse model which creates a positive feedback loop promoting tumor cell invasion and malignant progression via TGF-β 18. Thus, further exploration is required to unveil the multicellular interactions and spatiotemporal relevance of TGF-β signaling during the progression and metastasis of CC.

Single-cell RNA sequencing (scRNA-seq) has emerged as a powerful tool for characterizing TME and dissecting intratumor heterogeneity 19. Several scRNA-seq studies predominantly focused on investigating the diversity and heterogeneity of TME in primary CC or CC initiation 20-24. However, how tumor cells crosstalk with other important cell types to reprogram the TME of CC in both temporal and spatial dimensions during progression remain unclear. In this study, we integrated scRNA-seq profiling of localized, regional, and metastatic TME to elucidate the dynamic alterations in the proportion and the biological function of different cellular components. We identified a TGF-β signaling-responsive malignant subset, as well as pro-tumor neutrophils and cancer-associated fibroblasts (CAFs) that secreted TGF-β. These cells were found to be enriched in metastatic TME. Combining scRNA-seq data with spatial transcriptomics (ST) data further elucidated the colocalization and cell-cell interactions between these subsets. In addition, we provided a clustering strategy based on bulk RNA-seq that related these key clusters with metastasis and patients' prognosis.

## Materials and methods

### Data collection

The raw scRNA-seq data were obtained from our previously deposited database (the GSA human genome sequence archive: HRA001742 and HRA007492). Primary CC patients receiving no anti-tumor treatment at the time of sampling were included, patients with secondary CC or associated with other primary tumors were excluded. Detailed sources and clinical information of the patients in the scRNA-seq cohort are listed in Table S1. Our cohort included three localized primary tumors, four regional primary tumors and four metastatic primary tumors of CC. The processed spatial transcriptomics data were obtained from Science Data Bank (https://doi.org/10.57760/sciencedb.11624), published by Fan *et al.* 21. Normalized The Cancer Genome Atlas (TCGA) expression matrix and clinical information for cervical squamous cell carcinoma and endocervical adenocarcinoma (CESC) cohorts were obtained from the cBioPortal website (http://www.cbioportal.org/).

### scRNA-seq data preprocessing

Sequencing data were aligned to the GRCh38 human reference genome and then processed into the initial Unique Molecular Identifier (UMI) matrix using the Cell Ranger Single-Cell toolkit (version 7.0.1). The Seurat workflow (version 4.2.1) was utilized for downstream analyses with default parameters unless otherwise stated. Quality control measures were implemented to exclude cells with fewer than 1000 UMIs or fewer than 500 features or a mitochondrial gene fraction greater than 25%. The NormalizeData function was then used to normalize the library size and log-transform the expression matrix to prepare it for further downstream analyses.

### Data integration and cluster identification

Two rounds of unsupervised clustering were performed to identify major cell types and subclusters, and to eliminate residual doublets. The FindVariableFeatures function was used to identify the highly variable genes (HVGs). After scaling the data, Principal Component Analysis (PCA) was performed using the RunPCA function with the top 2000 HVGs for dimensionality reduction. For visualization, Uniform Manifold Approximation and Projection (UMAP) was implemented to reduce dimensionality in the RunUMAP function with the top 20 principal components. As we observed batch effects between patients in the UMAP plot, we used the canonical correlation analysis (CCA) function to integrate expression data from different samples for batch effect correction.

Cell clusters were annotated based on cluster-specific marker genes found by the FindAllMarkers function. Any clusters expressing more than two canonical cell type markers were considered as contaminated doublets and removed. Repeated dimension reduction and unsupervised clustering was performed if any doublets were removed. The first round of cell clustering and annotation identified major cell types including T and natural killer (T/NK) cells, myeloid cells, epithelial cells, B/plasma cells and stromal cells. To further analyze these major cell types, T/NK cells, myeloid cells, epithelial cells, B/plasma cells and stromal cells were reintegrated, re-clustered and re-annotated using the procedure described above.

### Distributed preference analysis

To characterize the tissue distribution of different clusters, odds ratios (OR) were calculated to indicate preferences as previously reported 25. Fisher's exact test was employed to obtain the OR and the corresponding p-value. P-values were adjusted using the Benjamini & Hochberg (BH) method with the R function *p.adjust*. An OR > 1.5 with an adjusted p-value < 1e-10 indicated a preference for cluster to be enriched in tissue, whereas an OR < 0.5 suggested an avoidance of distribution in tissue.

### Copy number variation (CNV) analysis

The CNVs of epithelial cells were assessed based on their transcriptome profiles using the infercnv method (version 1.14.2), which is designed to infer copy number alterations from tumor single-cell RNA-seq data. This package compares the expression intensities of genes across malignant cells and relates this to expression in normal cells. Fibroblasts were used as normal reference.

### Pathway enrichment analysis

The pathway scores for each cluster were calculated using the Gene set variation analysis (GSVA) method in GSVA package (version 1.46.0) 26. Additionally, the clusterProfile package (version 4.6.0) from Gene Set Enrichment Analysis (GSEA) was employed to show the enriched Hallmarks signaling pathways of each cluster. Human hallmark gene lists (h.all.v7.2.symbols.gmt) was downloaded from the GSEA website (https://www.gsea-msigdb.org/gsea/index.jsp).

To assess the potential function of the selected gene list and markers from the clusters, Gene ontology (GO) enrichment analysis was performed 27. This approach allowed us to examine the functional profiles and identify those significant differentially expressed genes (DEGs) that met the thresholds of |logFC|>0.50 and p < 0.05. The DEGs from each cluster were passed on to the clusterProfiler package for functional enrichment.

### Pseudotime trajectory analysis

To infer potential cell lineage trajectories between diverse cell phenotypes, we used Monocle2 (version 2.26.0) to estimate the pseudotime of each cell using default parameter and DDR-Tree method 28. Heatmap was employed to display the significant pseudotime-dependent genes.

In order to determine the significant genes that drive the phenotypic transformation of neutrophils, GeneSwitches package was applied. GeneSwitches is a statistical framework based on logistic regression to find the set of genes that switch during the transition. We applied GeneSwitches to analyze the trajectory of neutrophils transitioning from state1 to state3 (bottom branch) and to identify transcription factors (TFs) responsible for driving the transformation 29.

### Intercellular interaction analysis

CellChat (version 2.1.2) was utilized to infer cell-cell communications and significant pathways between different lineage subpopulations 30. First, ligands and receptors that were significantly overexpressed in specific cell clusters were identified. The probability of interaction between two paired clusters was calculated based on the average expression levels of ligands in one subset and receptors in the other. The significance of inferred cell-cell communication was assessed by permutation testing, and interaction pairs with a P value of below 0.05 were selected for further evaluation of intercellular communication.

### Spatial transcriptomics data process

The SCTransform function in Seurat was used to normalize the processed data from previous study 21. To annotate non-tumor spots in ST sample, we mapped cell types from scRNA-seq data using CytoSPACE analysis. Spots were annotated by highest cell type proportion. To identify the Epi4 and Epi0 spots, the expression score of Epi4_MYC and Epi0_AGR2 was calculated by UCell and visualized by plot_density function of Nebulosa (version 3.20) 31. Tumor spots with UCell score higher than 0.1 were considered as Epi0-like spots for further analysis. The cell-cell interactions of ST samples were analyzed by CellChat with default parameter settings. The spatial.factors parameter was set to ratio = 1 and tol = 25.

### Multiplex Immunohistochemistry (mIHC) staining

The mIHC staining was performed according to the manufacturer's instructions and our previous study 22. Briefly, 4μm thick formalin-fixed paraffin-embedded (FFPE) sections were stained four times with primary antibodies for epithelial cells (anti-PanCK, 1:2000, Proteintech), neutrophils (anti-CD15, 1:200, Proteintech), fibroblasts (anti-ACTA2, 1:1000, Proteintech) and E-cadherin (1:1000, Proteintech), followed by tyramide signal amplification (TSA) staining kits according to the manufacturer's instructions. Slides were scanned using a ZEISS Axioscan 7 microscope slide scanner and analyzed using ZEN microscopy software (version 3.9).

### Analysis of TCGA data

In the TCGA CESC cohort, the clinical and survival outcomes of patients having bulk RNA sequencing were analyzed. A signature for Epi0_AGR2 was defined comprising genes that highly expressed by Epi0_AGR2. To test if a given signature can predict survival, we first computed the average expression of the signature in each patient based on the bulk RNA-Seq data. Next, we stratified the patients into two groups (“high” and “low” group) according to the cutoff point identified by surv_cutpoint function in the survminer R package (version 0.4.9), where “high” indicates that the patient might have higher infiltration of Epi0_AGR2, and “low” suggests the opposite. Survival analysis was then conducted using the survfit function from the survival package (version 3.4.0), aiming to compare the survival differences between the "low" and "high" groups. Finally, we plotted Kaplan-Meier survival curves using the R function ggsurvplot, and p-values were calculated using the log-rank test.

To examine the prognostic value of gene signatures derived from multiple subsets in scRNA-seq analysis, we divided TCGA patients into multiple groups using unsupervised hierarchical clustering, and performed survival analysis to correlate the patient's prognosis with different gene signatures. Table S2 summarizes the gene signatures used in the survival analyses. The immune infiltration status of TCGA samples was estimated by data from the TIMER2.0 database (http://timer.cistrome.org/) 32.

### Statistical analysis

All statistical analyses were conducted using R statistical software (version 4.2.2). The data analysis followed the statistical methods described in the figure legends.

## Results

### Landscapes of cell composition in CC TME along tumor progression

**Figure 1:**
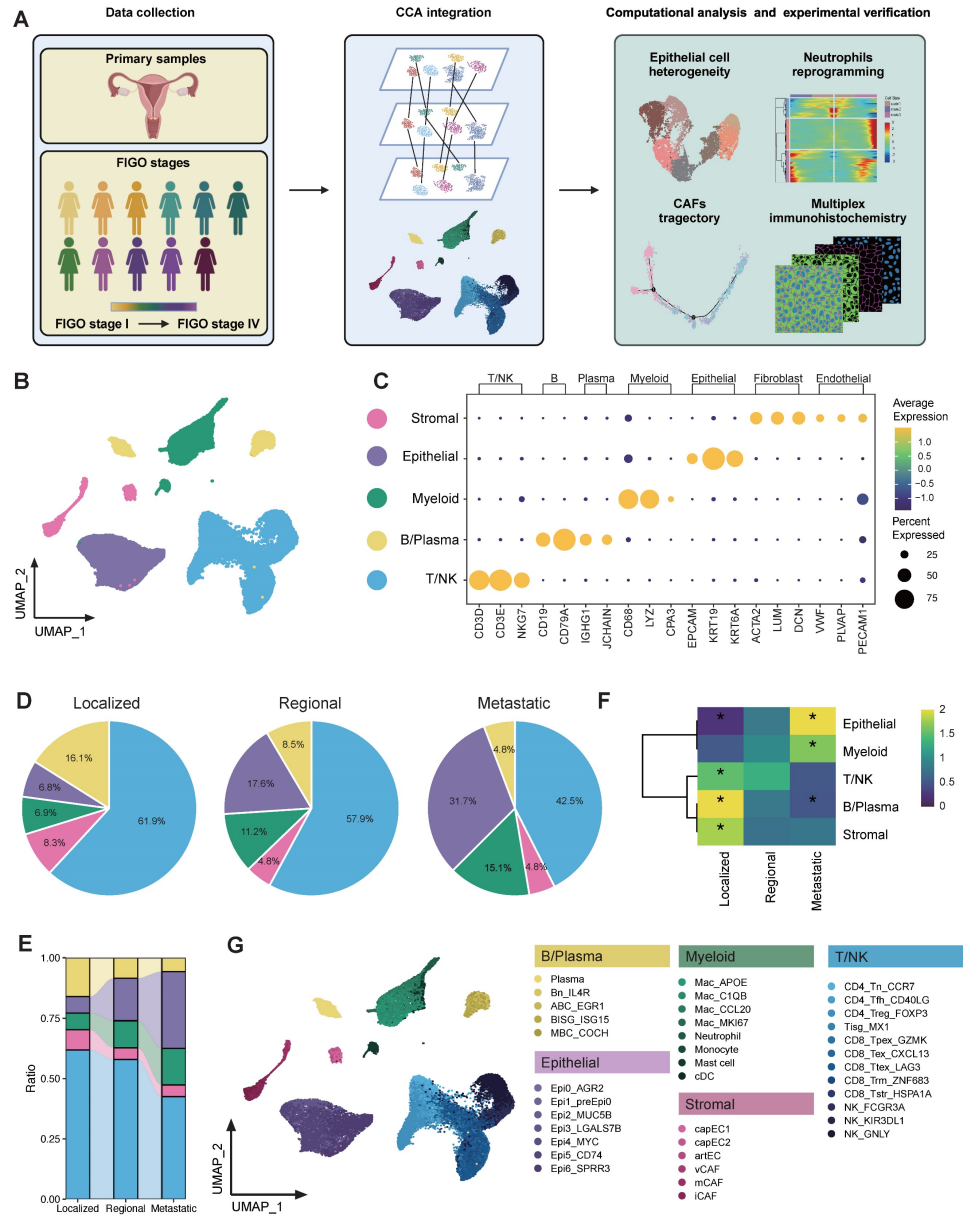
** Global analysis of tumor microenvironment in CC patients across different metastatic statuses. (A)** Scheme of the overall experimental design for the scRNA-seq analysis of 11 CC patients with different stages. **(B)** Uniform Manifold Approximation and Projection (UMAP) plot of the major cell types (denoted by colors). **(C)** Dot plots showing the expression of marker genes of major cell lineages. Dot size represents the fraction of cells expressing the given gene, with colors indicating the normalized expression levels. **(D)** A Pie chart presenting the proportion of each cell type in the 3 tissue origins. The cell cluster colors are identified in Fig. 1B. **(E)** Sankey plot illustrating the proportion of 5 major cell types among 3 groups. The cell cluster colors are identified in Fig. 1B. **(F)** Heatmap showing the ORs of major cell lineages occurring in each group. *OR > 1.5 indicates significant enrichment of the subset in the corresponding tissue; *OR < 0.5 indicated that it was preferable not to distribute in in the corresponding tissue. **(G)** UMAP plot of 38 subtypes (denoted by colors).

To obtain a comprehensive understanding of the cellular composition and molecular features during cervical cancer progression, we conducted scRNA-seq analysis on 11 primary tumor samples from CC patients diagnosed with differential pathological stages according to the FIGO (International Federation of Gynecology and Obstetrics) staging classification 33. Lesions of three patients were confined strictly to the cervix, and thus were categorized as localized group (corresponding to 2018 FIGO stage I). Four patients were classified as regional group, for their cancer had extended beyond the cervix to involve nearby pelvic structures, such as the upper portion of the vagina or parametrium (corresponding to 2018 FIGO stage II). The remaining four patients were classified as metastatic group, corresponding to 2018 FIGO stages IIIC-IV. This group included individuals whose tumors had either metastasized to distant organs or lymph nodes, signifying advanced disease progression (Figure 1A).

Following stringent data quality control and filtering (see methods), we obtained single-cell transcriptomes for 52,884 cells with 1,940 expressed genes detected per cell (Table S3), including 12,575 cells from localized tumors, 24,583 cells from regional tumors and 15,726 cells from metastatic tumors (Figure 1B and Figure S1A). CCA was performed in order to eliminate the batch effect, after which cells were subjected to clustering via the use of the UMAP. Five major cell populations were annotated according to canonical lineage markers, which comprised 10,162 epithelial cells, 28,710 T/NK cells, 5,011 B/Plasma cells, 6,001 myeloid cells, and 3,000 stromal cells (Figure 1C). The major cell lineages exhibited different cellular compositions across various groups. The relative abundance of epithelial cells and myeloid cells exhibited a progressive increase as CC tissues advanced from localized to regional and ultimately to metastatic groups (Figure 1D-E). Conversely, there was a gradual decrease in B/Plasma cells and T/NK cells. It was also found that cellular compositions were roughly similar across each individual within a group (Figure S1B). However, patient 1 (P1) was an exception to this due to the relatively small number of cells observed. The distribution preferences of major cell clusters across various group specimens were further examined through OR analysis (Figure 1F). OR analysis also confirmed the sparse infiltration of B/Plasma cells and T/NK cells in the metastatic group, revealing an immunosuppressive TME in metastatic CC. Additionally, the down-sampling analysis suggested that the clustering results were reproducible and not influenced by the total number of cells from each group (Figure S2A-B). A detailed investigation was further conducted into the sub-clustering of each major cell lineage, which resulted in the identification of 38 specific subsets (Figure 1G). These were identified on the basis of their unique expression profiles. Totally, we comprehensively delineated the cellular landscape at both the major lineage and specific subset levels in single cell resolution, and revealed the alterations in proportion along CC progression.

### The TME of metastatic group is indicative of the exclusion and exhaustion of CD8T cells

**Figure 2:**
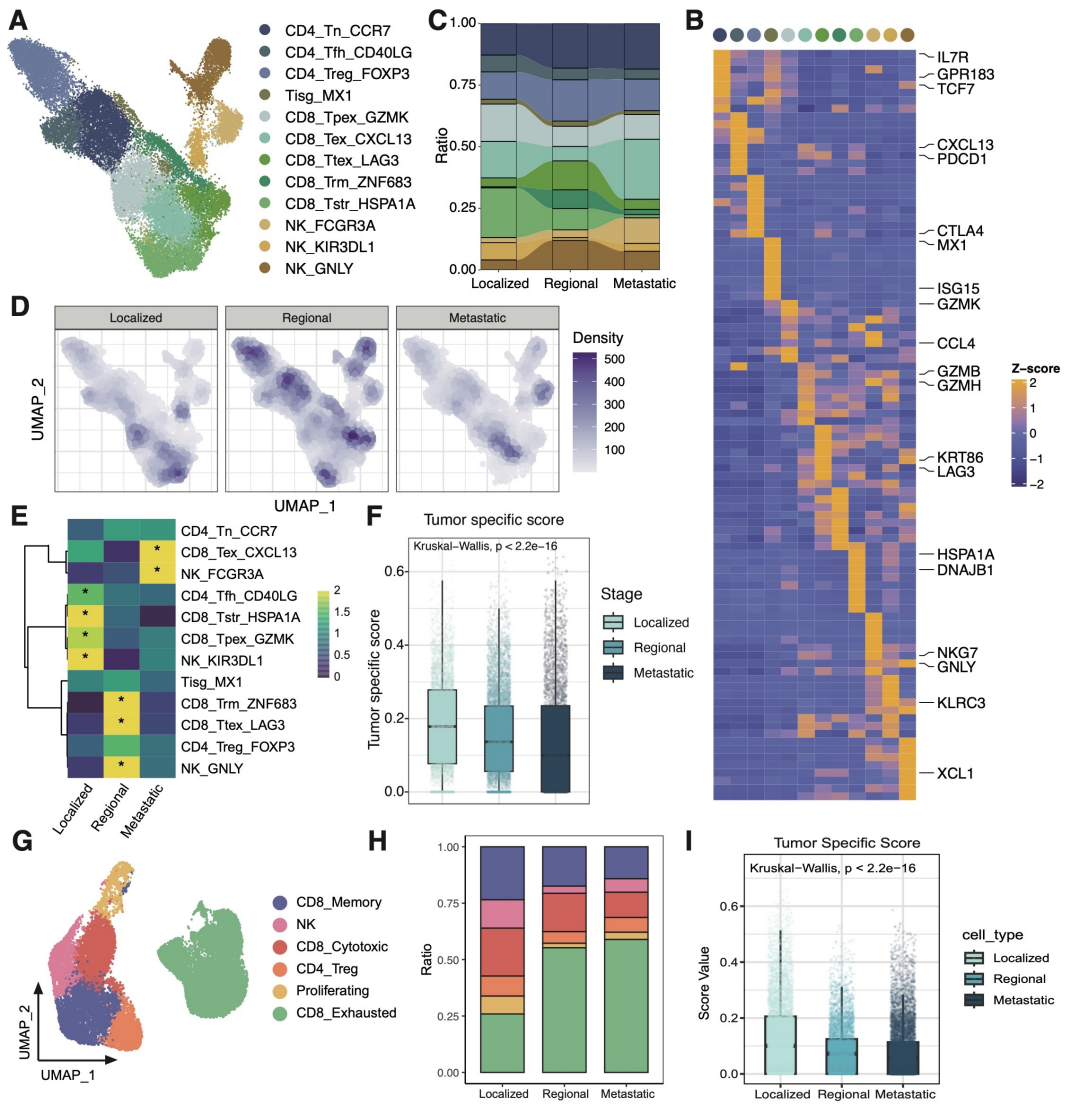
** scRNAseq analysis of T cells among 3 groups. (A)** UMAP plot of the T cell subsets (denoted by colors). **(B)** Heatmap showing marker genes of each T cell cluster. **(C)** Sankey plot illustrating the alteration in cellular composition of T cells among 3 groups. The cell cluster colors are identified in Fig. 2A. **(D)** UMAP density plots characterizing the distribution of T cells across 3 groups. **(E)** Heatmap showing the ORs of T cell subsets occurring in each group. *OR > 1.5 indicates significant enrichment of the subset in the corresponding tissue. **(F)** Boxplots showing the tumor specific score of T cells in 3 groups. Statistical significance was analyzed using Kruskal-Wallis test. The boxplots display the median, upper quartile, and lower quartile. **(G)** UMAP plot of the T cell subsets in external validation dataset (denoted by colors). **(H)** Stacked bar graph of the proportion of T cell cluster in each group in external validation dataset. The cell cluster colors are identified in Fig. 2G. **(I)** Boxplots showing the tumor specific score of T cells in 3 groups in external validation dataset. Statistical significance was analyzed using Kruskal-Wallis test. The boxplots display the median, upper quartile, and lower quartile.

The characteristics of T/NK cells were further examined across different groups (Figure 2A). Three CD4 T cell subsets, five CD8 T cell subsets, one interferon gamma related T cell subset, and three NK cell subsets were identified based on their markers as previously reported (Figure 2B) 22, 25. The metastatic group was characterized predominantly by an exhausted subcluster (CD8_Tex_CXCL13), implying that most tumor-infiltrating T cells in metastatic group are depleted and damaged due to long-term chronic antigen stimulation (Figure 2C-E). Besides, the tumor specificity score of CD8T cells in the metastatic TME was relatively low, indicating that the CD8T cells had a higher proportion of bystander T cell clones (Figure 2F) 34. In order to confirm the reliability of the research results, we analyzed immune microenvironment in an external validation data containing 16 CC patients 23, 35. It was also found that the proportion of exhausted T cells increased in the metastasis group, and the tumor-specific score decreased at the same time (Figure 2 G-I). Collectively, these observations highlight the presence of a markedly immunosuppressive microenvironment in the metastatic group, characterized by reduced T cell quantities, a shift towards an exhausted phenotype, and diminished tumor-specific recognition.

### Malignant cell subset Epi0_AGR2 was prevalent in metastatic group

**Figure 3:**
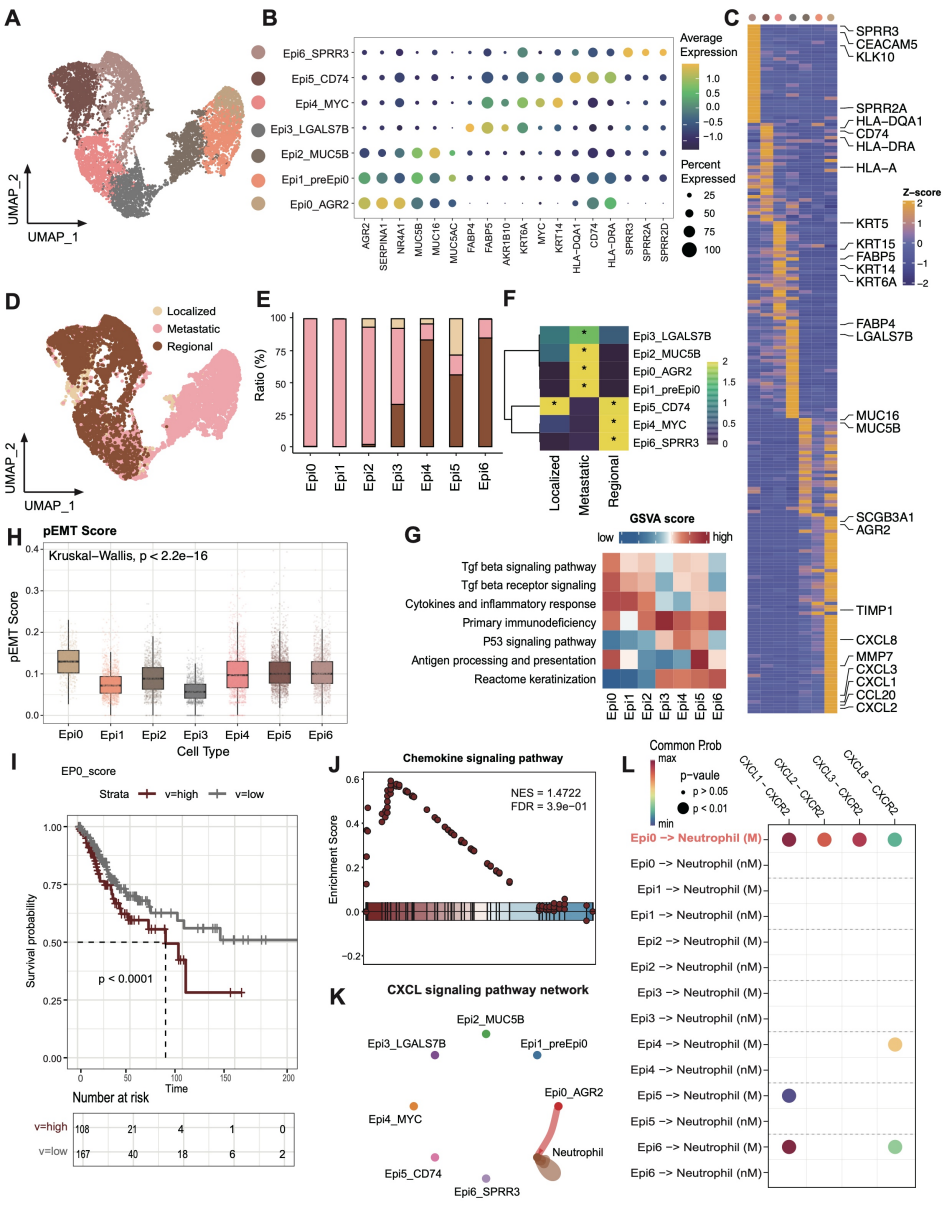
** scRNAseq analysis of malignant epithelial cells and identification of premetastatic sub-population. (A)** UMAP plot of the epithelial cell subsets (denoted by colors). **(B)** Dot plot showing the expression of marker genes of each epithelial cell subsets. Dot size represents the fraction of cells expressing the given gene, with colors indicating the normalized expression levels. **(C)** Heatmap showing marker genes of each epithelial cell cluster. **(D)** UMAP plot of different stages of the epithelial cell by color. **(E)** Stacked bar graph of the proportion of epithelial cell cluster in each stage. The stage colors are identified in Fig. 3D. **(F)** Heatmap showing the ORs of epithelial cell subsets occurring in each group. *OR > 1.5 indicates significant enrichment of the subset in the corresponding tissue. **(G)** Heatmap showing the GSVA score of phenotypic correlated pathways among epithelial subsets. **(H)** Boxplots showing the pEMT score of 7 epithelial cell clusters. Statistical significance was analyzed using KruskalWallis test. The boxplots display the median, upper quartile, and lower quartile. **(I)** Kaplan-Meier survival analysis of the EP0 signature in TCGA CESC cohort (n = 304). The survival curves were compared using the log-rank test. **(J)** GSEA analysis of Epi0_AGR2 subset. **(K)** Circle plots showing the CXCL signaling inferred by CellChat among epithelial cell and neutrophils. **(L)** Dot plot depicting the selected ligand-receptor interactions between epithelial cells and neutrophils. Communication probability and *P* values were calculated by CellChat, and were indicated by circle colour and size, respectively.

To investigate transcriptional heterogeneity and the TME alterations mediated by malignant cells in different metastatic statuses, we subdivided epithelial cell populations into seven subsets based on the expression of canonical marker genes and the top differentially expressed genes (DEGs) in each cluster (Figure 3A-B). No individual cluster was associated with a particular phase of cell cycle or with high proliferation (Figure S3A). According to the copy number variations (CNV) status, with fibroblasts as control group, all epithelial cells were judged to be malignant cells (Figure S3B). Epithelial cells exhibited a high extent of heterogeneity and showed a tendency to form clusters according to different groups in UMAP dimensions (Figure 3D-E). Concretely, Epi2_MUC5B represented adenocarcinoma cell (*MUC5B, MUC16, MUC5AC*), while Epi4_MYC harbored conventional squamous cell carcinoma features, such as high expression of *KRT6A* and *MYC* (Figure 3C). Epi3_FABP4 exhibited a gene expression module associated with lipid metabolism process (*FABP4*, *FABP5*, *AKR1B10*), showing low expression of major histocompatibility complex (MHC) I and MHC II, whereas Epi5_CD74 exhibited high expression of antigen presentation genes (Figure S3C). Epi6_SPRR3 exhibited epithelial differentiated features. Significantly, two hitherto unreported subsets, designated Epi0_AGR2 and Epi1_preEpi0, were identified, which exhibited joint characteristics and displayed elevated expression of multiple carcinogenic genes, such as *AGR2* and *NR4A1*, with exclusive enrichment in the metastatic group (Figure 3E-F). *AGR2* (encoding anterior gradient 2 protein) overexpression exhibited enhanced cancer cell proliferation and metastasis as well as promoting cells survival by increasing cancer cell fitness 36, 37.

### Transforming growth factor β signaling and chemokine signaling pathway are activated in Epi0_AGR2

To better understand the potential signaling pathways related to subsets of malignant cells, Gene Set Variation Analysis (GSVA) for hallmark gene sets was performed to validate the subtype annotation. Epi3_LGALS7B had higher gene activity scores for primary immunodeficiency. Epi5_CD74 had higher GSVA scores for antigen processing and presentation (Figure 3G), while Epi4 and Epi6 enriched in P53 and keratinization signaling pathway respectively, consistent with the dysfunction of P53 signaling in HPV related cancers 38. Importantly, we identified the TGF-β pathway to be only elevated in Epi0_AGR2 through GSVA analysis (Figure 3G). TGF-β responding tumor cells were previously characterized as a small subset with metastatic capacity, known as metastasis-initiating cells (MICs) 14, 39. In order to provide further confirmation of the MIC potential of Epi0_AGR2, the partial EMT (pEMT) score of various subsets was examined 40. The pEMT is widely considered to be of critical importance in tumor invasion and metastasis by maintaining the plasticity between epithelial and mesenchymal states 41. Epi0_AGR2 demonstrated a significantly higher pEMT score in comparison to the other subsets (Figure 3H). Furthermore, survival analysis of the TCGA CESC cohort (Figure 3I) revealed that patients exhibiting a high proportion of Epi0_AGR2 had a poor prognosis, thereby underscoring its MIC phenotype and its significance in CC progression.

In addition to the acquisition of mesenchymal traits by epithelial cells, EMT facilitates immune evasion in tumor cells, with the recruitment of immunosuppressive cells via the expressing of chemokines 42. In comparison with other subsets, Epi0_AGR2 was specifically expressed in a variety of EMT-related chemokines, particularly those associated with neutrophil chemotaxis, including C-X-C motif chemokine ligand 1 (CXCL1), CXCL2, CXCL3 and CXCL8 (Figure 3C) 42-44. GSEA analysis of Epi0_AGR2 subset also revealed significant upregulation of chemokine signaling pathway in comparison with other subsets (Figure 3J). To further verify the potential targets of these chemokines, cell-cell interaction analysis was conducted. Epi0_AGR2 demonstrated a specific interaction with neutrophils via the CXCL signaling pathway, and the interactions mediated by CXCR2 between tumor cells and neutrophils were found to be substantially enriched in the metastatic group (Figure 3K-L). Recently, the immune exclusion and dysfunction role of neutrophils were highlighted in a variety of cancers 45, 46, our results concluded that Epi0_AGR2 exhibited a significantly heightened capacity for neutrophil recruitment. We then analyzed the pEMT score and cytokine expression in tumor cells of different stages in the external validation dataset. The results suggested that tumor cells of advanced cervical cancer patients had higher pEMT scores and higher expression of a variety of neutrophil chemokines (Figure S3D-E). In summary, the present analysis identified the Epi0_AGR2 as TGF-β-responsive subsets with MIC characteristics, having the capacity for neutrophil recruitment and Epi0_AGR2 was found to be associated with a poor prognosis in CC.

### Inflammatory cytokine-enriched macrophages gathered in metastatic CC patients

**Figure 4:**
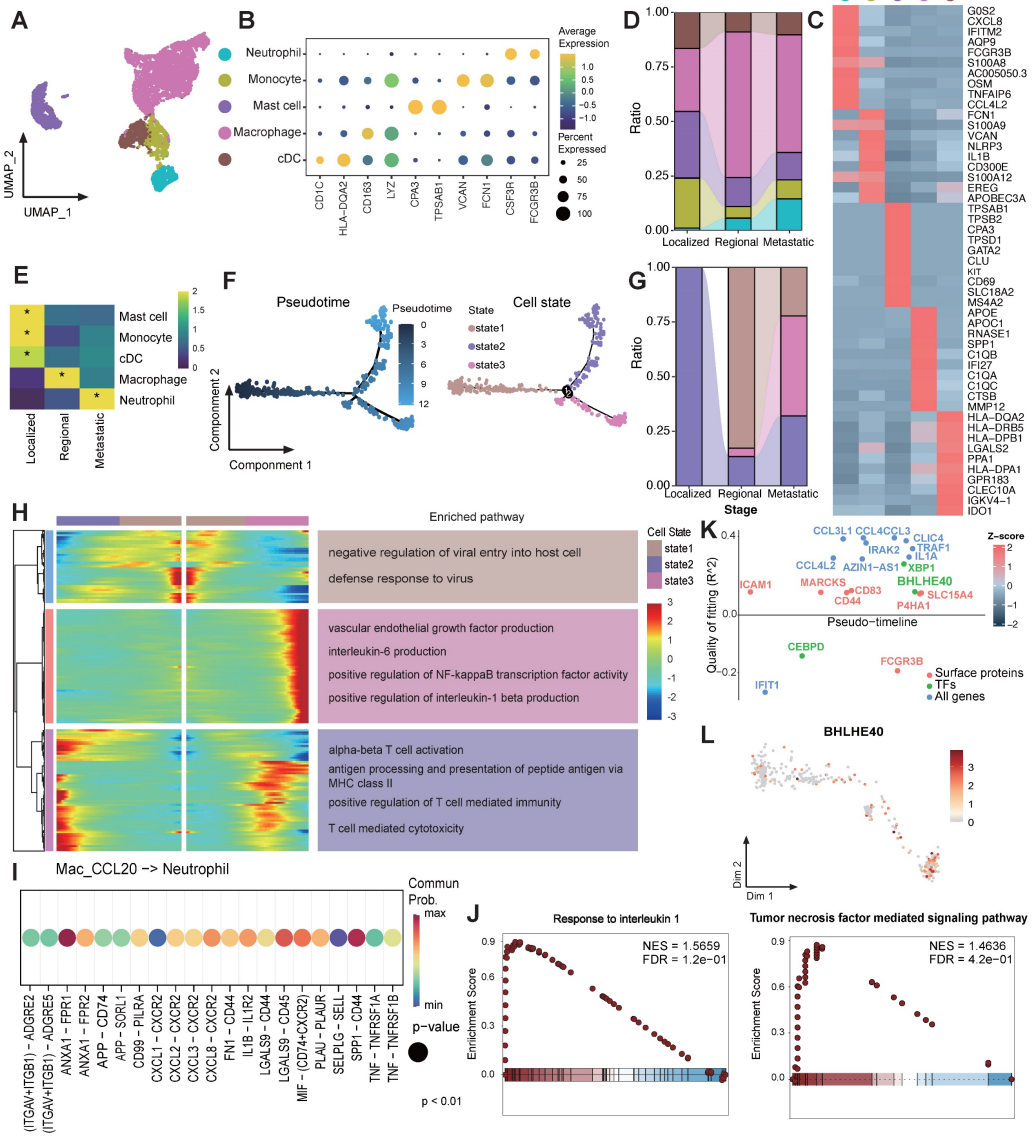
** Molecular profile of neutrophils during CC progression. (A)** UMAP plot of the myeloid-derived cells (denoted by colors). **(B)** Dot plot showing the expression of marker genes of each myeloid-derived cell subsets. Dot size represents the fraction of cells expressing the given gene, with colors indicating the normalized expression levels. **(C)** Heatmap showing marker genes of each myeloid-derived cell cluster. **(D)** Sankey plot illustrating the alteration in cellular composition of myeloid-derived cells among 3 groups. The cell cluster colors are identified in Fig. 4A. **(E)** Heatmap showing the ORs of myeloid-derived cell subsets occurring in each group. *OR > 1.5 indicates significant enrichment of the subset in the corresponding tissue. **(F)** Monocle 2 trajectory analysis of neutrophils. The trajectory was divided into three states indicated as state1, state2, and state3. **(G)** Sankey plot showing the distribution of neutrophils from different stages in different states. The state colors are identified in Fig. 4F. **(H)** Heatmap showing the dynamic DEGs and their enriched pathways along the trajectory. These DEGs were divided into three main clusters with different pathways enriched. **(I)** Dot plot depicting ligand-receptor interactions between neutrophils and Mac_CCL20 in CC tumour microenvironment. Communication probability and *P* values were calculated by CellChat, and were indicated by circle colour and size, respectively. **(J)** GSEA analysis of neutrophils in metastasis group. **(K)** Geneswitches output showing ordering of the top switching genes along the bottom neutrophil axis (State 1 to State 3). Key genes are highlighted with enlarged font size. **(L)** Expression plots of BHLHE40 of neutrophils on the bottom trajectory (state 1 to state 3).

We further conducted a comprehensive investigation of myeloid-derived cells within CC ecosystems, revealing the presence of 5 distinct subsets according to their classic markers (Figure 4A-C). Myeloid cells showed distinct preferences in distribution across different tumor stages. In early-stage tumors, monocytes, conventional dendritic cells (cDCs), and mast cells are the predominant myeloid-cell components, whereas macrophages dominated in advanced-stage tumors (Figure 4D-E). It is also noteworthy that the proportion of neutrophils progressively augmented during the course of tumor progression, in accordance with the accumulation of Epi0_AGR2 (Figure 4E). Consequently, the subsequent investigation concentrated on the macrophages and neutrophils.

Four distinct tumor-associated macrophages (TAMs) subsets were identified from macrophage populations (Figure S4A-B). All TAM clusters highly expressed M2-like macrophage gene signatures, angiogenesis-related genes and inhibitory immune checkpoint genes (Figure S4C), suggesting an immunosuppressive phenotype. Within the TAM clusters, Mac_APOE highly expressed C1Qs complement genes, implying its role in efferocytosis, which may promote immunosuppressive TME 47. Mac_C1QB expressed higher amounts of immediate early genes (*JUN*, *JUNB, JUND, FOS* and *EGR1*) encoding transcription factors, and may represent a subpopulation of transcriptionally active cells. The Mac_CCL20 subset, which exhibited similarities with inflammatory cytokine-enriched macrophages, was found to be enriched in metastatic group (Figure S4D-E). In order to gain further insights into the macrophage subset phenotypes in metastatic CC, a functional enrichment analysis was performed. This analysis revealed significant upregulation of pathways associated with angiogenesis, EMT, and NF-κB transcription factor (TF) activity in Mac_CCL20, while pathways related to antigen presentation and T cell activation was downregulated (Figure S4F). Interestingly, marked activation of “Neutrophil migration” and “Neutrophil chemotaxis” were noticed within Mac_CCL20 through GSEA analysis (Figure S4G), consistent with previous reported 48. Mac_CCL20 showed more interaction numbers with neutrophils in metastatic CC samples (Figure S4H), implying Mac_CCL20 possibly contributing to CC progression through promoting neutrophil recruitment. These findings suggest that high pro-metastatic and immunosuppression characteristics are critical identifiers of TAMs in metastatic group.

### Neutrophils accumulated and underwent significant transcriptional reprogramming during CC progression

It is demonstrated that various cell subsets, such as Epi0_AGR2 and Mac_CCL20, in metastatic CC are capable of recruiting neutrophils into TME. In order to further elucidate their role, an investigation was conducted into the contribution of neutrophils to the progression of CC. The pseudotime trajectory produced a prominent linear (left to right) trajectory with 2 distinct polarization directions (upper-state 2 and bottom-state 3), indicating 2 developmental fates of neutrophils both starting from state 1 (Figure 4F). Neutrophils from localized samples underwent upper trace to reach state 2, while neutrophils from metastatic patients were more likely to experience the bottom branch to reach state 3 (Figure 4G). To understand the transcriptional programs of neutrophils in developmental fates, we analyzed the differential gene expression profiles along the trajectory (Figure 4H). Neutrophils in state 2 are consistent with antigen-presenting cell-like neutrophils and are enriched in T cell activation and antigen presentation pathways 49, whereas state 3 was marked by pro-angiogenesis and inflammatory infiltration pathways, suggesting that neutrophil from differential stages have undergone various phenotypic changes. In light of the recognized functions of interleukin 1 beta (IL-1β) and IL6 in carcinogenesis, metastasis and immune escape 50, 51, it was inferred that neutrophils at state 3 exhibited a pro-metastatic phenotype.

To explore the drivers of neutrophil phenotypic transformation in metastatic group, CellChat was applied to infer cellular interactions. Mac_CCL20 stimulated neutrophils with pro-inflammatory cytokines IL-1 and tumor necrosis factor (TNF) (Figure 4I). Significant activation of TNF mediated signaling pathway and IL-1 signalling pathway were also observed in neutrophils of metastatic samples in comparison with others (Figure 4J). The Geneswitches algorithm was applied to the bottom branch (state1 to state3) to assess upstream TF driving the transformation of neutrophils in metastatic CC 29, which identified *BHLHE40* as potentially critical TF in this process (Figure 4K-L). *BHLHE40*, as a TF activated by hypoxia and endoplasmic reticulum (ER) stress, was considered as a key regulator driving neutrophils towards pro-tumor and immunosuppressive subtype 52, 53.

### Capillary endothelial cell subset capEC2 and vascular fibroblast vCAF are associated with tumor progression and immunosuppression

**Figure 5:**
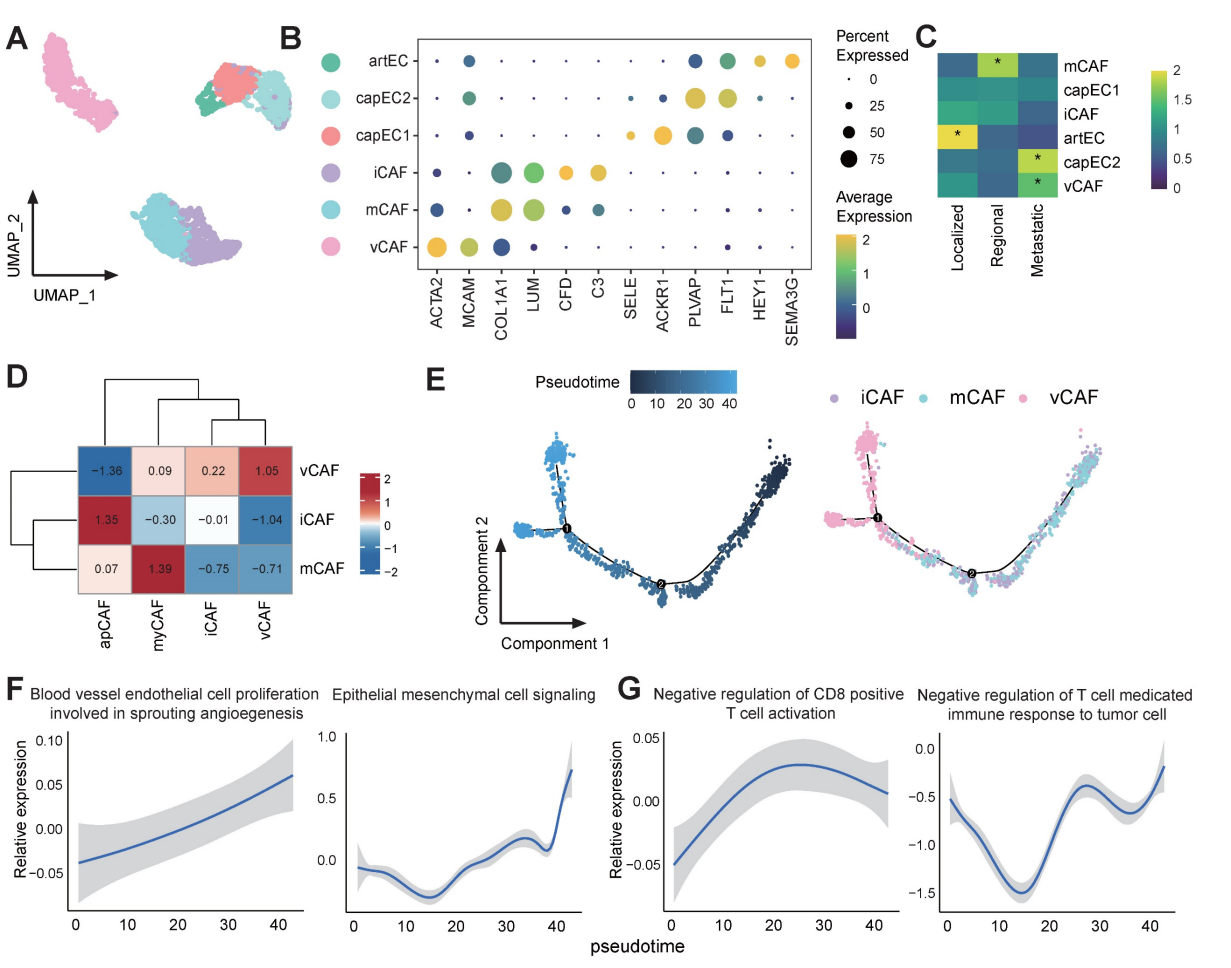
** Characterization of CAFs in metastatic CC. (A)** UMAP plot of the stromal subsets (denoted by colors). **(B)** Dot plot showing the expression of marker genes of each stromal subsets. Dot size represents the fraction of cells expressing the given gene, with colors indicating the normalized expression levels. **(C)** Heatmap showing the ORs of stromal subsets occurring in each group. *OR > 1.5 indicates significant enrichment of the subset in the corresponding tissue. **(D)** Heatmap showing expression of reported CAF gene signatures across CAFs clusters. **(E)** Monocle 2 trajectory analysis of CAFs. **(F-G)** GSEA analysis of of selected pathways along the pseudo-time.

Stromal cells in the tumor TME influence various processes during tumor development and influence how cancer cells respond to treatment 54. We next examined the population of stromal cells, including endothelial cells (ECs) and cancer associated fibroblasts (CAFs). Among the ECs, an arterial EC (artEC) subset and 2 capillary EC (capEC) subsets with distinct transcriptional features were identified (Figure 5A-B). It was observed that capEC2 displayed significant enrichment in metastatic tumors (Figure 5C), and the Gene Ontology (GO) term enrichment analyses indicated that capEC2 was enriched for epithelial cell migration and vascular endothelial growth factor receptor signalling pathway (Figure S5A). Furthermore, GSEA analysis of the capEC2 subset revealed significant upregulation of angiogenesis and epithelial cell migration signalling pathways, and downregulation of immunoactivation-related pathways in comparison with other EC subsets (Figure S5B-C). In addition, the capEC2 exhibited high expression of vascular endothelial growth factor receptor (VEGFR) genes FLT1 (also known as VEGFR1) and KDR (also known as VEGFR2) compared with other ECs (Figure S5D). Consequently, the capEC2 demonstrated augmented interaction strength with Epi0_AGR2 via the VEGF signalling pathways (Figure S5E-F), thereby suggesting the importance of capEC2 in the process of angiogenesis and tumor vascularization. This data elucidated the pro-metastatic properties and immunosuppressive phenotypes of capEC2.

CAFs in our study included inflammatory CAFs (iCAFs) which expressed high levels of genes involved in alternative complement activation pathway, such as C3 and CFD; matrix CAFs (mCAFs) highly expressed marker genes involved in the organization of extracellular matrix and vascular CAFs (vCAFs) subset which expressed angiogenesis genes (Figure 5A-B). vCAFs displayed significant enrichment in metastatic tumors (Figure 5C). The characters of CAFs were further verified by quantifying the expression of gene signatures of previous reported CAF subtypes (Figure 5D) 55. vCAF have been described as cancer-associated fibroblasts promoting angiogenesis and tumor progression 56. The pseudo-time analysis revealed that vCAF existed in the final stage of the cell trajectory (Figure 5E). Furthermore, it was evident that metastatic pathways, including "blood vessel endothelial cell proliferation involved in sprouting angiogenesis" and "epithelial mesenchymal cell signalling", as well as immunosuppressive pathways underwent a process of enrichment as the pseudo-time progressed (Figure 5F-G). These data suggested that vCAF differentiation was tightly connected with metastatic TME. Together, our results highlight considerable stromal changes along CC progression, cellular dynamics in capEC2 and vCAFs support a consistent phenotypic shift of stromal cells towards driving tumor immunosuppression and angiogenesis.

### OSM-OSMR signalling activated CAFs and enhanced the expression of TGF-β

**Figure 6:**
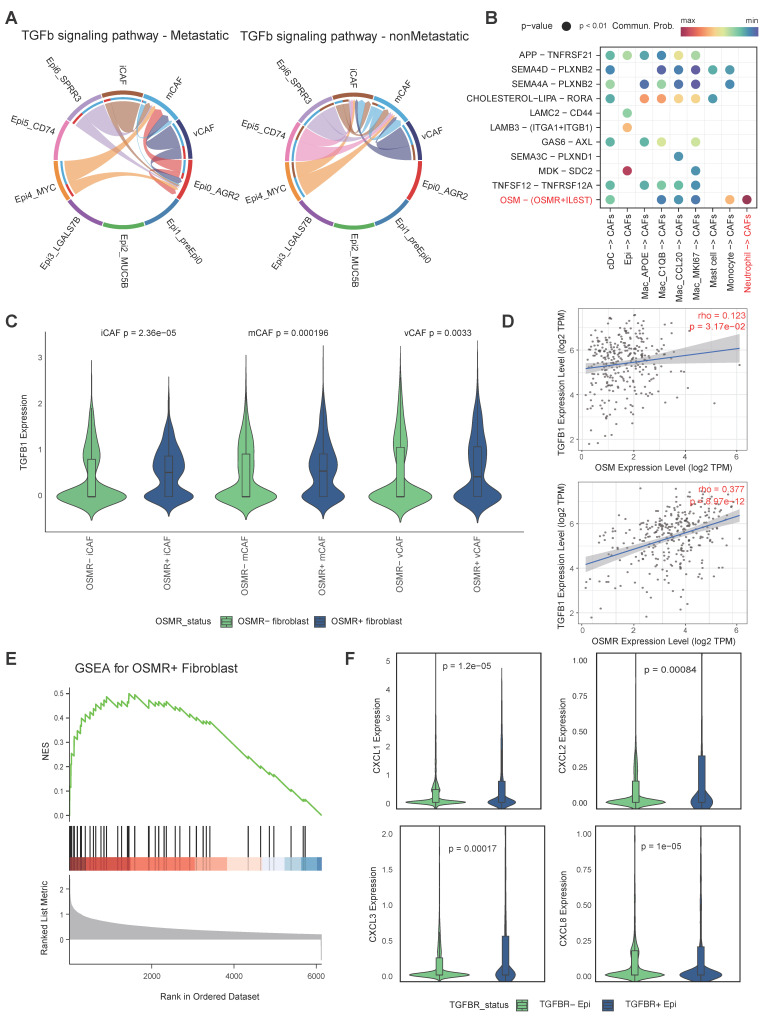
** Cell-cell interations among epithelial cells, neutrophils and CAFs. (A)** Circle plots showing the TGF-β signaling inferred by CellChat among epithelial cell and CAFs. **(B)** Dot plot depicting the ligand-receptor interactions between epithelial cells myeloid cells and CAFs. Communication probability and *P* values were calculated by CellChat, and were indicated by circle colour and size, respectively. **(C)** Violin plots showing expression level of TGF-β in CAFs subtypes according to positive or negitive OSMR expression. *P* values were determined using wilcox.test. **(D)** Correlation of OSM and OSMR levels with TGFB1 expression in CC samples. Spearman's correlation coefficients and *P* values are shown. TPM, transcript count per million reads. **(E)** GSEA showing enrichment of the indicated signatures in OSMRpos CAFs. NES, normalized enrichment score. **(F)** Truncated violin plots showing expression level of indicated chemotaxis in epithelial cells according to positive or negitive TGFBR expression. *P* values were determined using wilcox.test.

A mounting body of evidence indicates that the intricate interplay among tumor cells, CAFs 57-59, as well as myeloid cells 60-62 is instrumental in facilitating tumor progression and metastasis. Our present research has revealed the preference of Epi0_AGR2, neutrophils and vCAFs in metastatic group. Hence, we further investigated the interactions among these cell types along progression of CC via CellChat. As anticipated, heightened TGF-β signalling was observed between CAFs and Epi0_AGR2 in the metastatic group, indicating that CAFs potentially serve as the origin of TGF-β within the CC TME (Figure 6A) 63. Of significance, the investigation revealed a pronounced interaction of OSM-OSMR between neutrophils and CAFs (Figure 6B). Oncostatin M (OSM), a member of the IL-6 family, plays a pivotal role in the immunopathogenesis of various cancers, including CC 64, 65. Moreover, OSM is predominantly expressed by neutrophils, while OSMR is primarily expressed by stromal cells in CC (Figure S6 A-B). The TIMER database was utilised to analyse the association between OSM and OSMR expression and TME composition in the TCGA CESC cohort. The results revealed a significant association between OSM mRNA levels and neutrophils (Figure S6C), and between OSMR mRNA expression and CAFs enrichment (Figure S6D). These findings indicated that the OSM-OSMR signal is a specific interaction between neutrophils and fibroblasts in CC.

Further investigation into the relationship between OSMR expression and CAFs phenotype revealed elevated levels of TGF-β expression in OSMR^pos^ CAFs compared to OSMR^neg^ CAFs (Figure 6C). The correlation between OSM or OSMR and TGF-β expression in CC was further demonstrated by TIMER (Figure 6D). Furthermore, GSEA analysis showed that OSMR^pos^ CAFs had signatures related to JAK/STAT3 signaling, in agreement with increased STAT3 phosphorylation by OSM 66, which promoted CAF activation (Figure 6E). Collectively, these findings underscore the notion that OSM-OSMR signaling plays a pivotal role in CAF activation and TGF-β production.

### A positive feedback loop within pro-metastatic niche accelerating CC progression

Based on the elevated abundance of Epi0_AGR2, neutrophils and CAFs, as well as specific cellular interactions among these cell types, we propose a hypothesis that cellular interplay of three subsets formatted a positive feedback loop and promoting CC metastasis and progression. Epi0_AGR2 is a TGF-β responding tumor cell cluster, characterized by high expression of multiple chemotaxis and the ability to attract neutrophils to TME. We then wondered the association between TGF-β signaling and Epi0_AGR2 phenotype. Notably, CXCL1, CXCL2, CXCL3 and CXCL8 mRNA expression in epithelial cells is associated with TGFBR levels (Fig. 6F). TIMER analysis also certified that CXCL1, CXCL2 and CXCL3 mRNA expression is associated with TGFBR levels in CC patients (Figure S6E).

**Figure 7:**
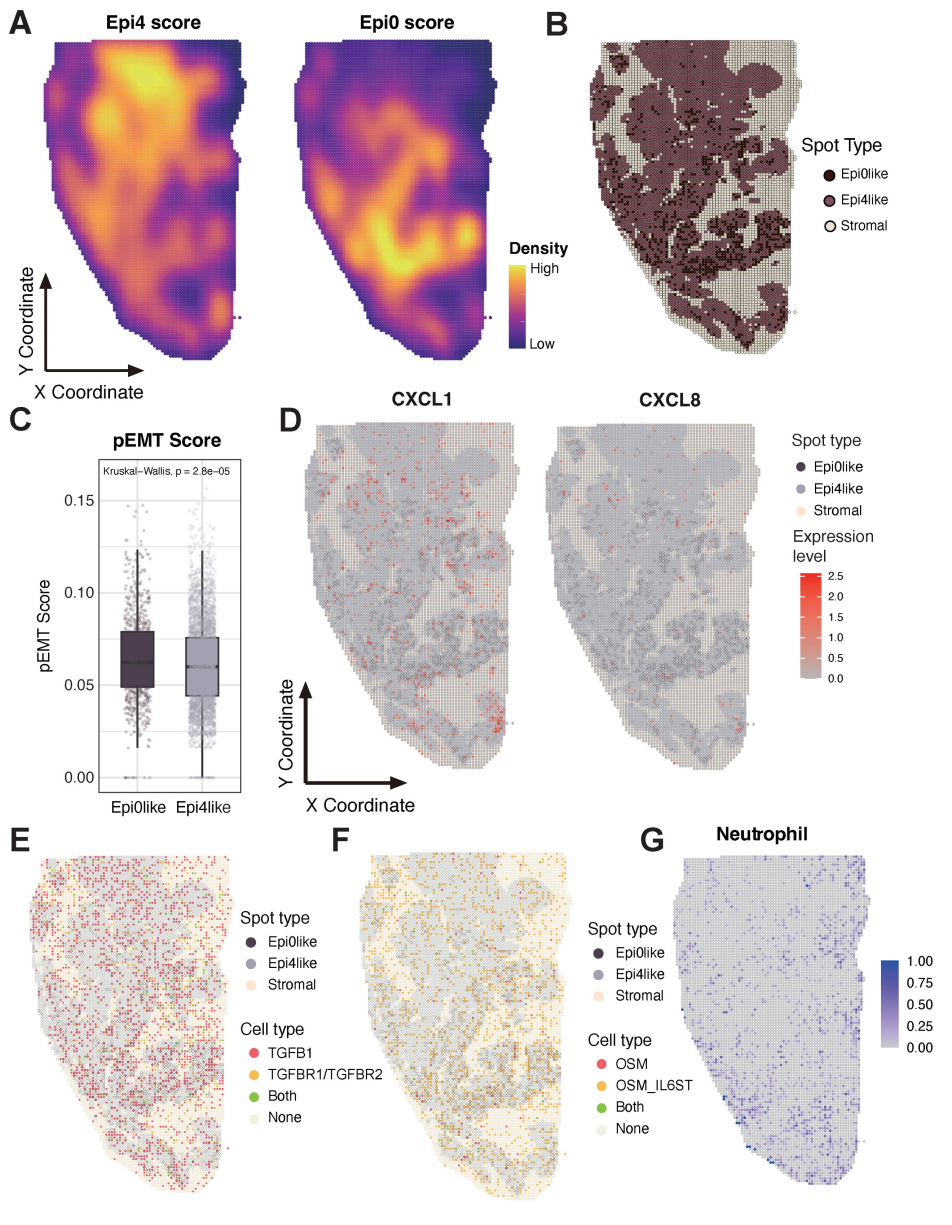
** Unique spatial distribution of Epi0 subset in ST sample, co-localizing with neutrophils and showing enhanced CXCL and OSM signaling pathways. (A)** Epithelial subset signatures in ST data. **(B)** The spot distribution of Epi0, Epi4 and stromal cells in ST data. Epi0, Epi4 and stromal subsets were identified by utilizing corresponding highest-scoring subset signatures from scRNA-seq data. **(C)** Boxplots showing the pEMT score of Epi0like and Epi4like spots identified in ST data. Statistical significance was analyzed using KruskalWallis test. The boxplots display the median, upper quartile, and lower quartile. **(D)** Expression levels of CXCL1 and CXCL8 in ST data. **(E)** The distribution of cells with different TGFB and TGFBR expression in ST data. **(F)** The distribution of cells with different OSM expression in ST data. **(G)** Neutrophils signatures in ST data.

The spatial colocalization of these cell types was then investigated, and a verification was conducted on a ST sample of squamous cell carcinoma (SCC) measuring 1.0*2.0 cm. The gene signatures of Epi0_AGR2 and the classic SCC subset Epi4_MYC were extracted to detect the characteristics of the tumor area in ST sample. The ST sample exhibited distinct Epi4-like and Epi0-like areas (Figure 7A). The Epi0_AGR2 score was predominantly concentrated in the lower half of the slide, particularly along the periphery, suggesting the potential invasive capacity of Epi0-like tumor spots. The tumor spots were further categorized as Epi0-like and Epi4-like according to their Epi0_AGR2 score level (Figure 7B). Epi0-like spots in ST also exhibited similar characteristics to the Epi0_AGR2 subset in scRNA-seq, such as higher pEMT signature score and higher expression of both CXCL1 and CXCL8 (Figure 7C-D). To further investigate the cell-cell interactions between tumor spots and other components, subsequent visualization of the ligand-receptor pairs of TGF-β and OSM-OSMR signaling on tissues was conducted. The results revealed that their distribution was concentrated on the Epi0-like spots and their adjacent spots, thereby supporting the elevated interactions of these interactions being located on the Epi0-like zone (Figure 7E-F). Additionally, the neutrophil proportion was found to be enriched in proximity to these regions (Figure 7G).

To systematically validate the predicted Epi0_AGR2-neutrophil-CAF interactions, parallel CellChat analyses were performed on both the scRNA-seq and ST datasets (Fig. S7A and C). A thorough examination of the intersection of ligand-receptor pairs common to both modalities revealed the presence of conserved signaling patterns. Notably, ANXA1 and MDK-mediated interactions among Epi0_AGR2, CAFs, and neutrophils exhibited strong concordance in both scRNA-seq and ST (Fig. S7B, and E). A notable finding was the observation that TGFβ signaling exhibited a conserved interaction strength between Epi0_AGR2 and fibroblasts/stromal cells across both platforms (Fig. S7C and F). This multi-modal validation reinforces confidence in the spatial co-occurrence and biological relevance of these interactions.

**Figure 8:**
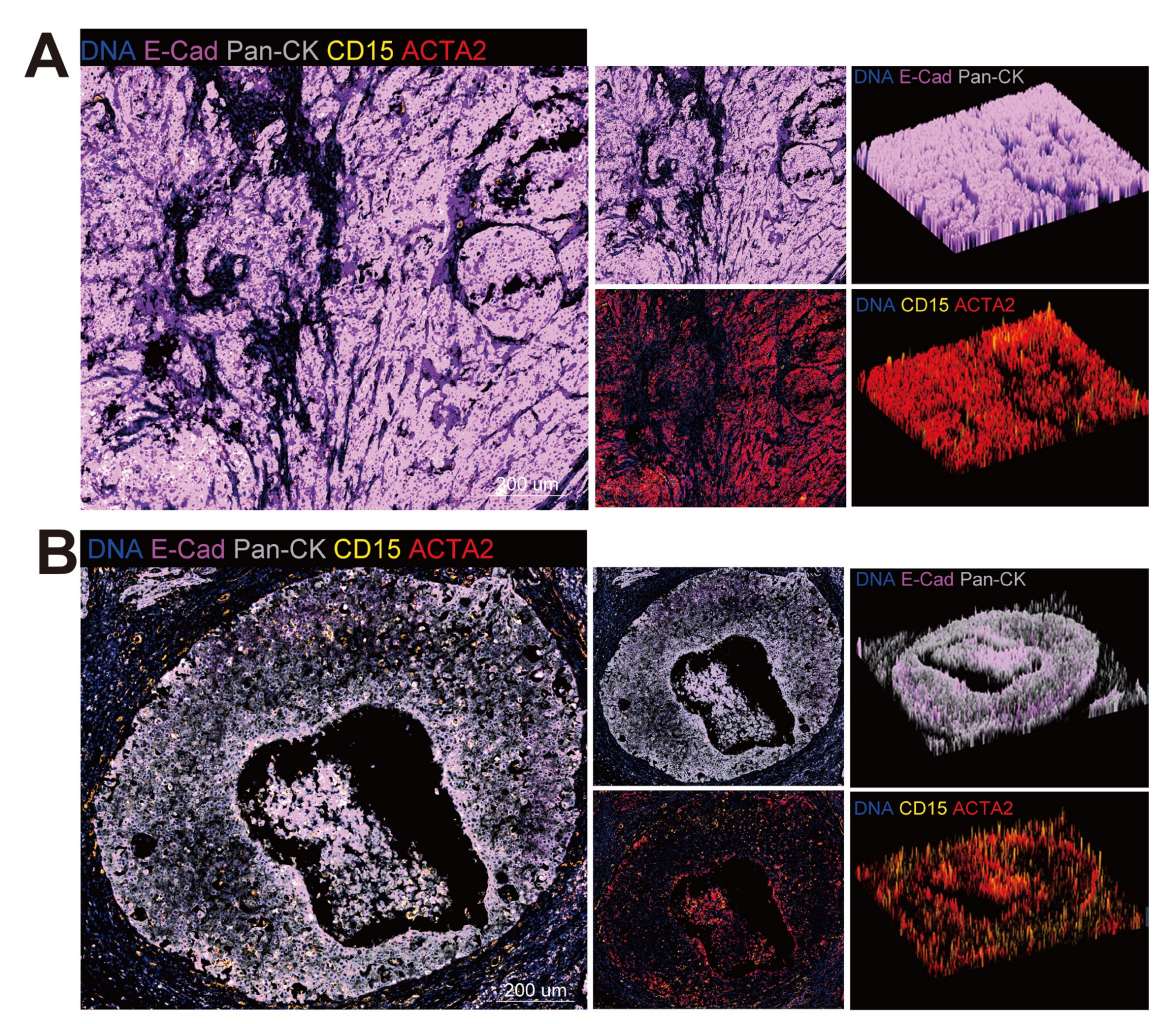
** Cell colocalization in different stages of CC. (A)** Epithelial cells, CAFs and neutrophils in center zone of the tumor area are exhibited by mIHC via differential markers as follow: epithelial cells: Pan-CK+, neutrophils: CD15+, CAFs: ACTA2+; Pan-CK+E-Cad- cells represent epithelial cells that are undergoing EMT. scale = 200μm. **(B)** Epithelial cells, CAFs and neutrophils in edge zone of the tumor area are exhibited by mIHC via differential markers as follow: epithelial cells: Pan-CK+, neutrophils: CD15+, CAFs: ACTA2+; Pan-CK+E-Cad- cells represent epithelial cells that are undergoing EMT. scale = 200μm.

Furthermore, the mIHC were conducted on the slide of a patient with stage IIIC disease (Figure 8A-B). By staining the epithelial marker pan-CK and E-cadherin, which is a marker of tight junctions, the tumor compartment and the extent of EMT could be distinguished, for the loss of E-cadherin was considered to be the hallmark of EMT 67. A comparison was made between centre zone (Figure 8A) and edge zone (Figure 8B) of the tumor area. Tumor cells in the edge zone exhibited a decrease in E-cadherin and were surrounded by neutrophils (CD15) and CAFs (ACTA2). The data obtained from this study suggest the existence of a niche consisting of Epi0_AGR2, neutrophils and CAFs, where a positive feedback loop among cancer cells, neutrophils and CAFs promotes tumor progression. Epithelial cells secrete cytokines, inducing the aggregation of neutrophils, which, in turn, activate CAFs through OSM-OSMR signalling, thereby inducing the MIC phenotype of cancer cells through the TGF-β signalling pathway.

### Clinical relevance of the pro-metastatic niche

**Figure 9:**
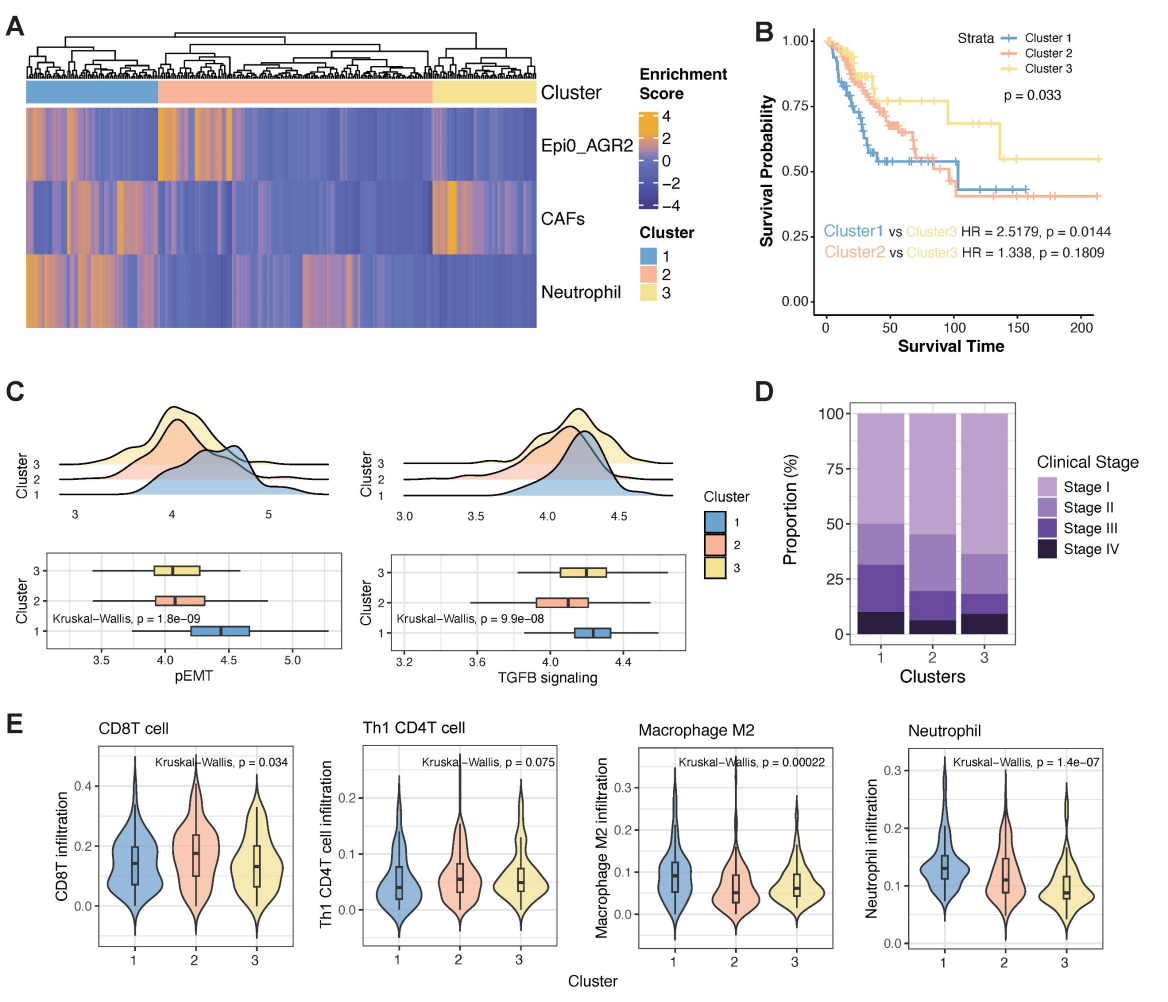
** Co-occurrence of epithelial cells, CAFs and neutrophils predicts poor outcomes for TCGA CC patients. (A)** Unsupervised hierarchical clustering for patients from the TCGA dataset based on the correlation to Epi0_AGR2, CAFs and neutrophils. **(B)** Comparison of overall survival (OS) rates for three clusters identified in Fig. 9A. *P* values are calculated using the log-rank test. **(C)** Histogram showing the proportion of different clinical stages in three clusters identified in Fig. 9A. **(D)** Boxplots showing the pEMT and TGFB_signaling scores of three clusters identified in Fig. 9A. Statistical significance was analyzed using Kruskal-Wallis test. The boxplots display the median, upper quartile, and lower quartile. **(E)** Violin plots showing the score of different cell signatures in three clusters identified in Fig. 9A. Statistical significance was analyzed using Kruskal-Wallis test.

In determining the prevalence of the co-occurrence of Epi0_AGR2, CAFs and neutrophils in CC patients and their potential clinical relevance, we utilized an unsupervised hierarchical clustering approach to examine the co-occurrence of Epi0_AGR2, CAFs and neutrophils in the TCGA database. As demonstrated in Figure 9A, a joint model incorporating these cellular components offered a promising prognosis model for CC patients. Patients in cluster 1 exhibited a strong co-occurrence of Epi0_AGR2, CAFs and neutrophils, suggesting that they harbored the pro-metastatic niche and consequently had a higher risk of death (HR = 2.5179, p = 0.0144, Figure 9B). In contrast, the lack of the co-occurrence of all three cell types showed a relative better survival prognosis. The gene signature of pEMT and the TGF-β signalling pathway exhibited elevated gene activity in samples from cluster 1, thereby confirming the characteristics of the pro-metastatic niche in patients from this cluster (Figure 9C). Consequently, a higher proportion of patients in cluster 1 were found to be in advanced stages (Figure 9D). The findings provide a compelling rationale for the correlation between the co-occurrence of these cell types and a worse clinical outcome in CC patients, and support the potential for the model to serve as a reliable prognostic tool. Furthermore, an in-depth investigation was conducted into the infiltration of immune cells in samples of differential clusters. The cluster 1 showed lower infiltration of anti-tumour immune cells such as CD8 T cells and Th1-like CD4 T cells, whereas higher infiltration of pro-immune evasion cell types such as M2 macrophages and neutrophils in comparison with other clusters (Figure 9E). Collectively, these observations imply that the pro-metastatic niche may also be susceptible to the immunosuppressive TME of CC in advanced stages, and could serve as a viable target for combination therapy involving ICIs.

## Discussion

Tumors can be considered as evolving multicellular ecosystems, with the interactions among various cell components in TME contributing collectively to tumor progression 68. In this study, we comprehensively characterized the tumor, myeloid, and stromal components within CC at the single-cell resolution and spatial level. Distinct molecular features and specific cellular communities associated with metastatic CC were identified. The intra-tumoral heterogeneity of cancer cells was revealed, and Epi0_AGR2, a subset of malignant cells that secrete multiple cytokines and chemokines, was identified as a potential MIC subtype. Furthermore, the enrichment of Mac_CCL20 subclusters and neutrophils with an immunosuppressive phenotype in metastatic CC, and their function, was highlighted. Of particular significance was the description of a pro-metastatic niche that exhibited a positive feedback loop, promoting CC progression through multi-omics analysis and validation.

It is imperative to recognize the significance of chemokine secretion by tumor cells, for those chemokines are key regulators of TME by not only inducing proliferation, invasion, metastasis of tumor cells, but also altering the tumor immunity and therapeutic outcomes via influencing different immune cell subsets 69. A recent study found that a stem-like osteosarcoma cell cluster producing CXCL14 to form a lung metastatic niche, further highlighting the role of chemokines are not limited in TME 70. It is evident that the existence of chemokine-dependent positive feedback loops can lead to the enhancement of initial small differences in anti- and pro-tumorigenic responses over time 71. Hence, it is vital to identify chemokines that may promote tumor progression, metastasis and immunosuppression, and to elucidate their regulatory mechanisms. In this study, we identified a significant upregulation of the chemokines CXCL1, CXCL2, CXCL3 and CXCL8 in the Epi0_AGR2 subset. These chemokines were hypothesized to be responsible for the recruitment of neutrophils. However, further investigation is required to elucidate the regulatory mechanisms underlying these chemokines. On the one hand, a autocrine feedback loop has been identified between EMT and chemokines such as CXCL2 and CXCL8. In certain types of cancer, the EMT phenotype and the signalling of these chemokines are associated with same TFs, such as SNAIL 72, 73. On the other hand, the employment of small molecular inhibitors and antibodies directed against both CXCL8 and its receptor CXCR2, which is also a receptor for the chemokines CXCL1 and CXCL2, has been explored in the context of clinical trials investigating cancer therapies, thereby underscoring the potential for clinical applications 74. Consequently, the characteristics of chemokine secretion may be potential vulnerabilities and therapeutic targets for Epi0_AGR2 cells.

The characteristics of neutrophils remain to be extensively investigated, despite the evident neutrophilia exhibited by CC patients and the substantial neutrophil infiltration present in CC cases, which is associated with a poor prognosis 75-77. Emerging evidence underscores the multifaceted role of neutrophils in shaping an immunosuppressive TME and driving cervical cancer progression 46. Beyond their canonical immune functions, tumor-associated neutrophils (TANs) can directly promote tumor cell invasion through protease secretion and neutrophil extracellular trap (NET)-mediated ECM remodeling. Critically, NET can orchestrate immune evasion via arginase-1-driven T-cell exhaustion 78. While our current study highlights neutrophils synergistically with CAFs secreting TGF-β via OSM/OSMR signaling. This bidirectional crosstalk establishes a feed-forward loop, suggesting spatially coordinated immunosuppression. Future work integrating neutrophil depletion models and single cell cytokine profiling will be essential to dissect the hierarchy of these interactions. Targeting the neutrophil-CAF metabolic coupling via CXCR2 inhibition may represent a novel strategy to disrupt this axis.

The spatial organization of cellular communities within the cervical TME critically determines functional output, a dimension our study acknowledges but warrants deeper mechanistic exploration. For example, Fibroblast-enriched invasive margins physically segregating cytotoxic CD8+ T cells may establish immunological exclusion zones that facilitate metastatic dissemination 79. Of clinical relevance, spatially resolved ligand-receptor analysis revealed tumor cells at the stromal interface preferentially expressing fibronectin-binding integrins (ITGA5/ITGB1) 80, and targeting integrin α5 in fibroblasts potentiates colorectal cancer response to PD-L1 blockade 81. Future studies employing multiplexed ion beam imaging or CODEX could resolve how pro-metastatic niches encode global TME dysfunction, informing spatially targeted therapies.

There are several limitations of our study. First, while the analysis demonstrates robustness within the current sample cohort, the conclusions require validation through larger-scale single-cell datasets to enhance generalizability. Secondly, although pro-metastatic niches were identified through clinical specimen profiling, their functional roles in metastasis remain to be elucidated mechanistically. These refinements will serve to enhance the biological relevance and therapeutic applicability of our findings.

## Conclusions

This study comprehensively characterized the TME within CC of different stages at single-cell resolution, showing a significant Immunosuppressive microenvironment in metastatic CC and uncovered subsets that play key roles in tumor progression. Besides, we discovered and validated a pro-metastatic niche comprising MIC like tumor cells subset Epi0_AGR2, neutrophils and CAFs, that exhibited a positive feedback loop, promoting CC progression through multi-omics analysis.

## Supplementary Material

Supplementary figures and tables.

## Abbreviations

BH: Benjamini & Hochberg
CAFs: Cancer-Associated Fibroblasts
CNV: Copy Number Variation
DEGs: Differentially Expressed Genes
ECs: Endothelial Cells
EMT: Epithelial-Mesenchymal Transition
ER: Endoplasmic Reticulum
FLT1: Fms-Related Tyrosine Kinase 1
GO: Gene Ontology
GSEA: Gene Set Enrichment Analysis
GSVA: Gene Set Variation Analysis
HR: Hazard Ratio
HPV: Human Papillomavirus
ICIs: Immune Checkpoint Inhibitors
IL-1β: Interleukin-1 beta
IL-6: Interleukin-6
iCAFs: inflammatory Cancer-Associated Fibroblasts
JAK/STAT3: Janus Kinase/Signal Transducer and Activator of Transcription 3
KDR: Kinase insert Domain-Containing Receptor
MICs: Metastasis-Initiating Cells
mCAFs: matrix Cancer-Associated Fibroblasts
MHC: Major Histocompatibility Complex
NET: neutrophil extracellular trap
OSM: Oncostatin M
OSMR: Oncostatin M Receptor
OR: Odds Ratio
PCA: Principal Component Analysis
pEMT: partial Epithelial-Mesenchymal Transition
PMN-MDSCs: Polymorphonuclear Myeloid-Derived Suppressor Cells
TAMs: Tumor-Associated Macrophages
TANs: tumor-associated neutrophils
TF: Transcription Factor
TME: Tumor Micro-environment
TNF: Tumor Necrosis Factor
TIMER: Tumor Immune Estimation Resource
UMAP: Uniform Manifold Approximation and Projection
VEGF: Vascular Endothelial Growth Factor
VEGFR: Vascular Endothelial Growth Factor Receptor
vCAFs: vascular Cancer-Associated Fibroblasts
