# Supplementary material for: Multi-Omics Analysis Reveals the transforming growth factor-β Signaling-Driven Multicellular Interactions with Prognostic Relevance in Cervical Cancer Progression
Source: J Cancer. 2025 Jun 20;16(9):2857–76. doi: 10.7150/jca.114505 (PMC12244011; doi:10.7150/jca.114505)
Supplement: Supplementary file 1 — Supplementary figures and tables. [file jcav16p2857s1.pdf]

Supplementary Figure

Figure S1. Heterogeneity of cell composition among patients.

A) UMAP plot colored by patient identity number. Localized patients: CC1, CC3, and CC6. Regional patients: CC4, CC7, CC10 and CC11. Metastatic patients: CC2, CC5, CC8 and CC9.

B) Sankey plot illustrating the proportion of 5 major cell types in 11 CC patients. The cell cluster colors are identified in Fig.1B.

Figure S2. Analysis of TME landscapes, Related to Figure 1.

A) UMAP plots showing the TME cell clusters and cell densities across different tissue groups with all cells included.

B) Down-sampling analysis showing the results are reproducible and not influenced by the total number of cells from each group.

Figure S3. Characterization of epithelial cell subsets.

A) UMAP plot showing average z-score expression of cell cycle gene signatures.

B) Heatmap shows CNVs for tumor subsets with the reference of fibroblasts. The results were inferred by inferCNVpy.

C) Dot plots showing the MHC class I and II gene expression of epithelial cell subsets.

D) Boxplots showing the pEMT score of epithelial cells in 3 groups in external validation dataset. Statistical significance was analyzed using Kruskal-Wallis test. The boxplots display the median, upper quartile, and lower quartile.

E) Dot plots showing the chemokine gene expression of epithelial cell in 3 groups in external validation dataset.

Figure S4. The phenotype diversity of macrophage.

A) UMAP plot of the macrophage (denoted by colors).

B) Dot plot showing the expression of marker genes of each macrophage subsets. Dot size represents the fraction of cells expressing the given gene, with colors indicating the normalized expression levels.

C) Expression levels and frequencies of genes composing the M1-like, M2-like, angiogenesis and checkpoint genes across myeloid cell clusters.

D) Heatmap showing the ORs of myeloid-derived cell subsets occurring in each group. *OR > 1.5 indicates significant enrichment of the subset in the corresponding tissue.

E) Violin plot showing the expression of cytokines for each macrophage subset. Colors represent different cell subsets.

F) GSEA enrichment plot showing the upregulated pathways (blue) and downregulated pathways (green) of Mac_CCL20 compared to other macrophage subsets.

G) GSEA analysis of Mac_CCL20 subset.

H) Circle plots show cell-cell interaction number differences of macrophage and neutrophils

in localized, regional and metastatic CC samples.

Figure S5. Endothelial cell transcriptomes altered during CC metastasis.

A) The enriched GO terms of EC subclusters. a. lymph vessel morphogenesis.

B) GSEA analysis showing the upregulated pathways of capEC2 compared to other EC subsets.

C) GSEA analysis showing the downregulated pathways of capEC2 compared to other EC subsets.

D) Differentially expressed genes of capEC2.

E) Circle plots showing the VEGF signaling inferred by CellChat among epithelial cell and ECs.

F) Dot plot depicting ligand-receptor interactions between epithelial cell and ECs. Communication probability and *P* values were calculated by CellChat, and were indicated by circle colour and size, respectively.

Figure S6. Additional analysis of figure 6.

A) Violin plot showing the expression of OSM for each cell subset. Colors represent different cell subsets.

B) Violin plot showing the expression of OSMR for each cell subset. Colors represent different cell subsets.

C-D) Correlation of OSM (C) and OSMR (D) expression with infiltration level of indicated cell types in CC samples. Spearman's correlation coefficients and *P* values are shown. TPM, transcript count per million reads.

E) Correlation of TGFBR1 and TGFBR2 levels with CXCL1, CXCL2 and CXCL3 expression in CC samples. Spearman's correlation coefficients and *P* values are shown. TPM, transcript count per million reads.

Figure S7. Additional analysis of figure 7.

A) Circle plots depicting cell-cell interaction strength from CellChat analysis of scRNA-seq dataset.

B) Bubble plots of conserved ligand-receptor pairs shared between scRNA-seq modalities. Bubble size indicates interaction probability.

C) Circle plots depicting cell-cell interaction strength from CellChat analysis of ST dataset.

D) Bubble plots of conserved ligand-receptor pairs shared between ST modalities. Bubble size indicates interaction probability.

E-F) Heatmaps comparing TGFβ signaling interaction strengths in (E) scRNA-seq and (F) ST data.

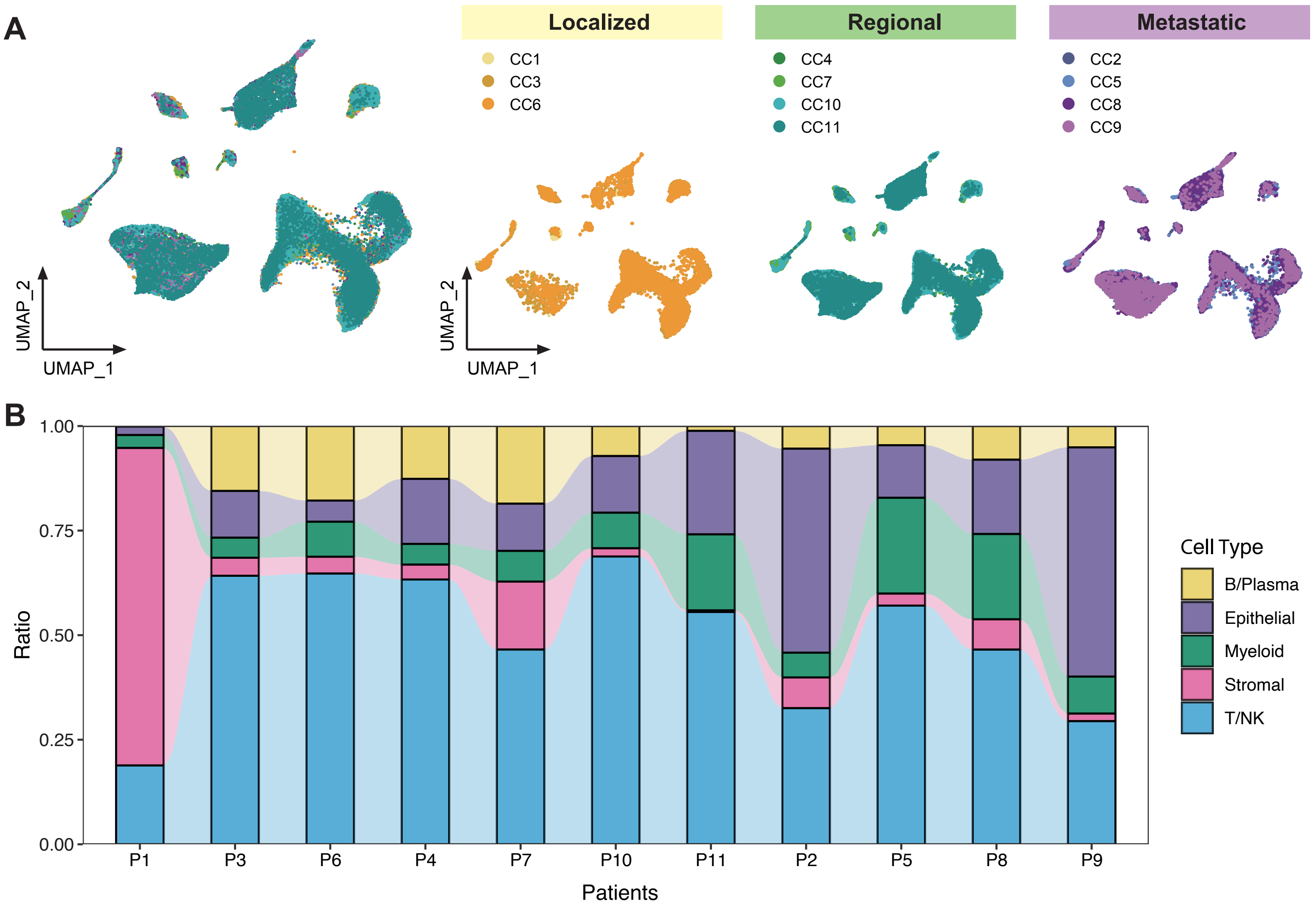
A
Localized
Regional
Metastatic
CC1
CC3
CC6
CC4
CC7
CC10
CC11
CC2
CC5
CC8
CC9
UMAP_2
UMAP_1
UMAP_2
UMAP_1
B
Ratio
1.00
0.75
0.50
0.25
0.00
P1
P3
P6
P4
P7
P10
P11
P2
P5
P8
P9
Patients
Cell Type
B/Plasma
Epithelial
Myeloid
Stromal
T/NK

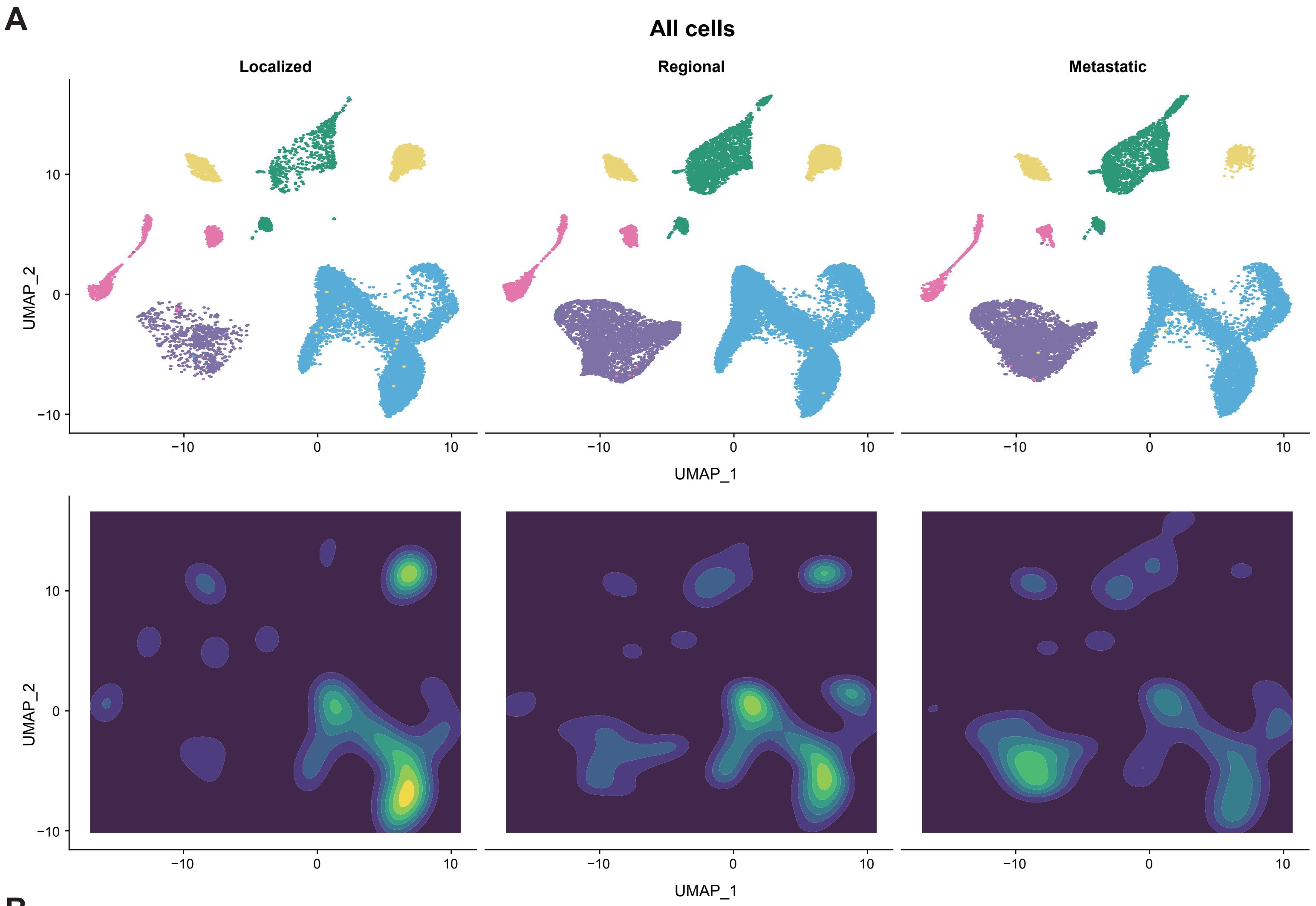
A
All cells
Localized
Regional
Metastatic
UMAP_2
UMAP_1

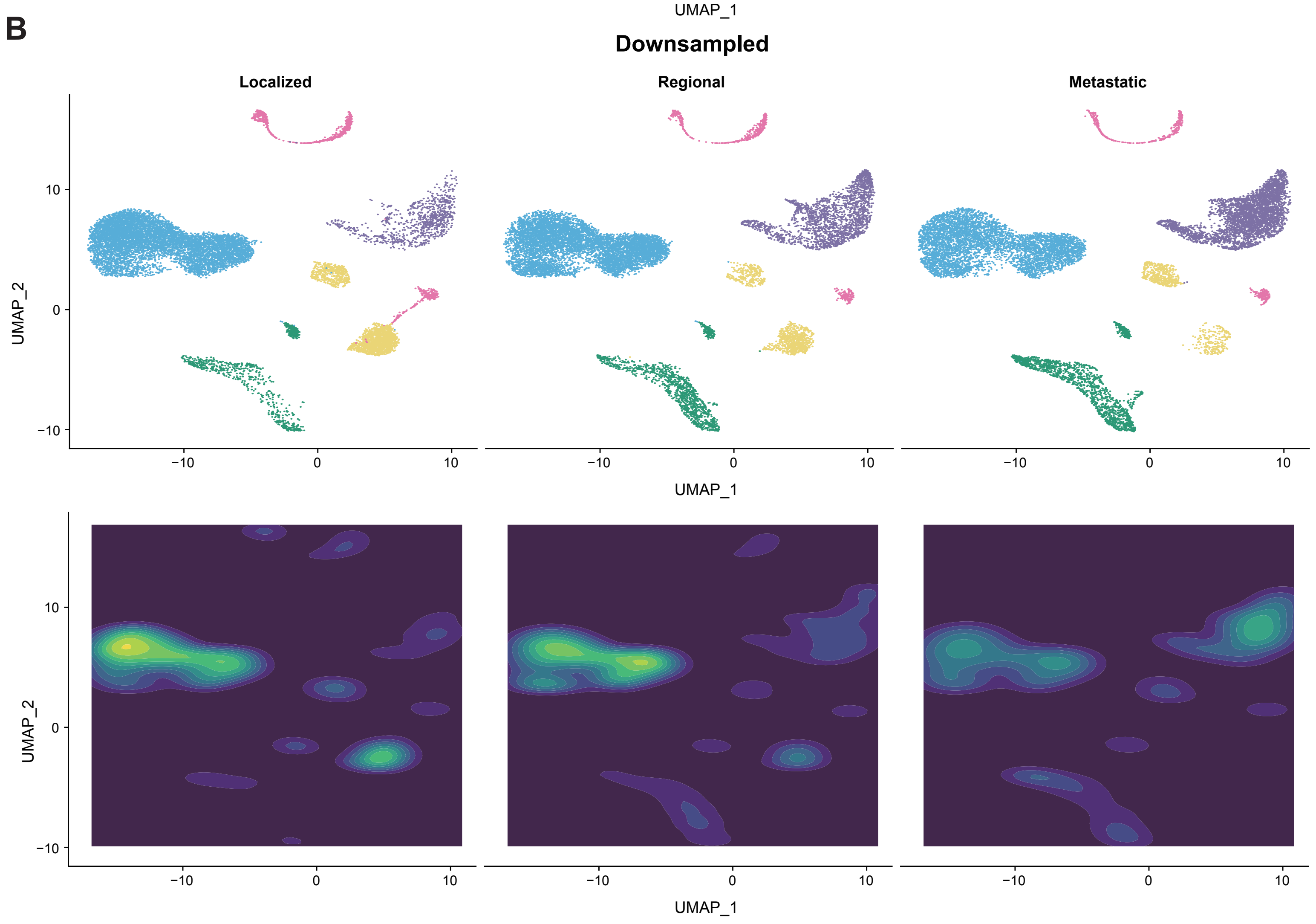
B
Downsampled
Localized
Regional
Metastatic
UMAP_2
UMAP_1

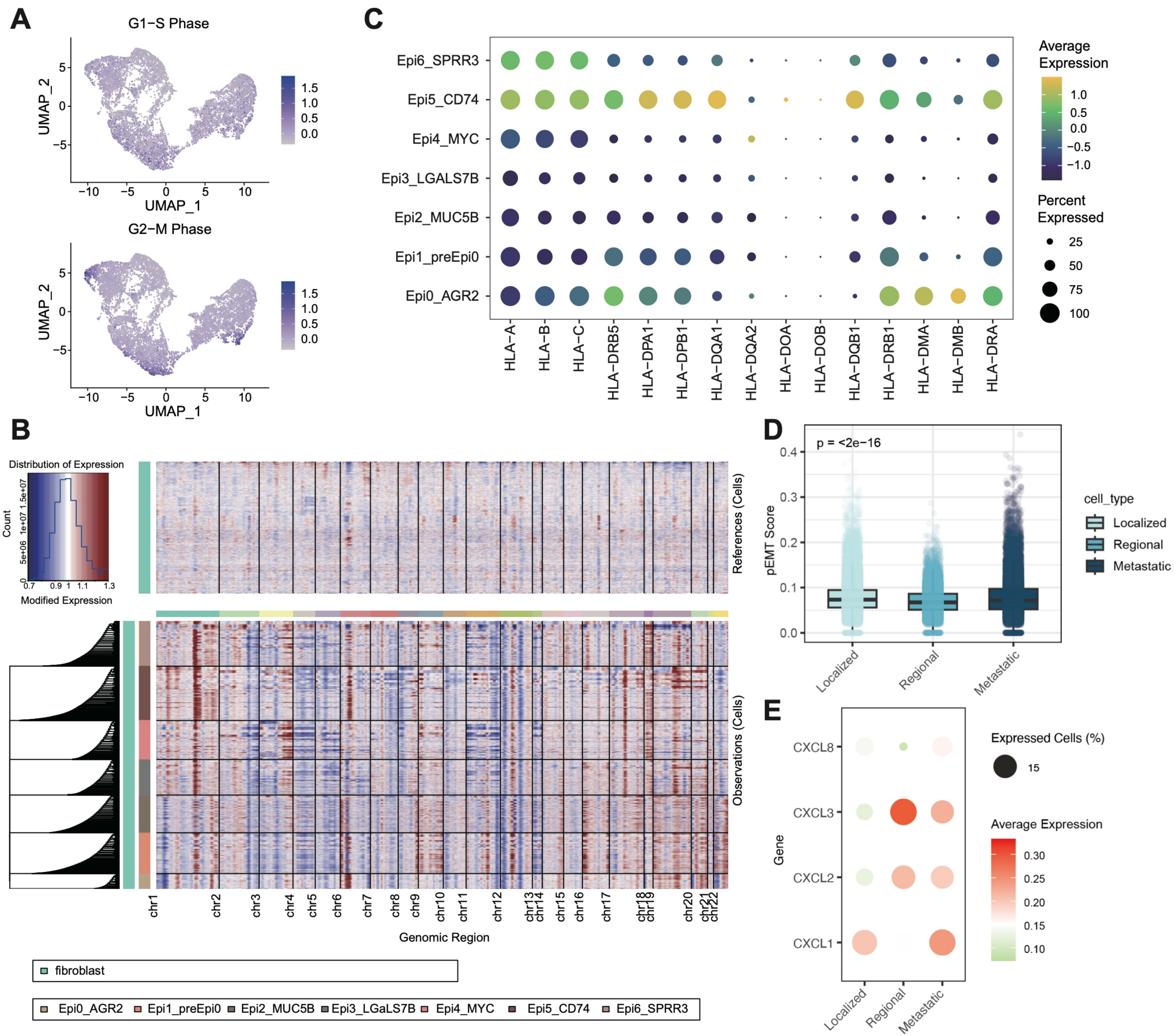

A
G1−S Phase
UMAP_2
UMAP_1
G2−M Phase
B
Distribution of Expression
Count
Modified Expression
References (Cells)
Observations (Cells)
Genomic Region
fibroblast
Epi0_AGR2
Epi1_preEpi0
Epi2_MUC5B
Epi3_LGaLS7B
Epi4_MYC
Epi5_CD74
Epi6_SPRR3
C
Epi6_SPRR3
Epi5_CD74
Epi4_MYC
Epi3_LGALS7B
Epi2_MUC5B
Epi1_preEpi0
Epi0_AGR2
HLA−A
HLA−B
HLA−C
HLA−DRB5
HLA−DPA1
HLA−DPB1
HLA−DQA1
HLA−DQA2
HLA−DOA
HLA−DOB
HLA−DQB1
HLA−DRB1
HLA−DMA
HLA−DMB
HLA−DRA
Average Expression
Percent Expressed
D
p = <2e−16
pEMT Score
cell_type
Localized
Regional
Metastatic
E
Gene
CXCL8
CXCL3
CXCL2
CXCL1
Expressed Cells (%)
Average Expression

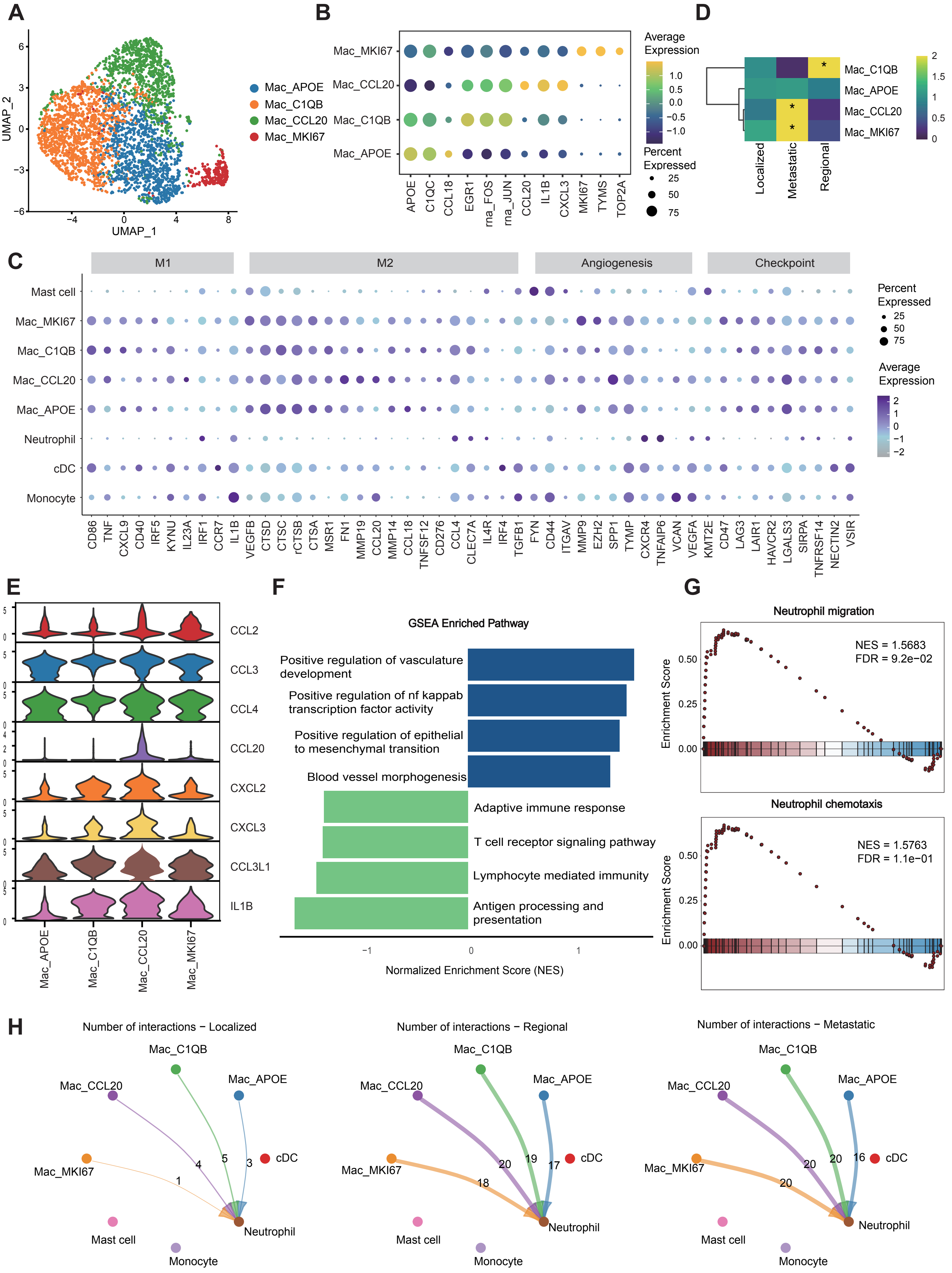
A
B
C
D
E
F
G
H
UMAP_1
UMAP_2
Mac_APOE
Mac_C1QB
Mac_CCL20
Mac_MKI67
Average Expression
Percent Expressed
Localized
Metastatic
Regional
M1
M2
Angiogenesis
Checkpoint
Mast cell
Neutrophil
cDC
Monocyte
CCL2
CCL3
CCL4
CCL20
CXCL2
CXCL3
CCL3L1
IL1B
GSEA Enriched Pathway
Positive regulation of vasculature development
Positive regulation of nf kappab transcription factor activity
Positive regulation of epithelial to mesenchymal transition
Blood vessel morphogenesis
Adaptive immune response
T cell receptor signaling pathway
Lymphocyte mediated immunity
Antigen processing and presentation
Normalized Enrichment Score (NES)
Neutrophil migration
NES = 1.5683
FDR = 9.2e−02
Neutrophil chemotaxis
NES = 1.5763
FDR = 1.1e−01
Enrichment Score
Number of interactions − Localized
Number of interactions − Regional
Number of interactions − Metastatic

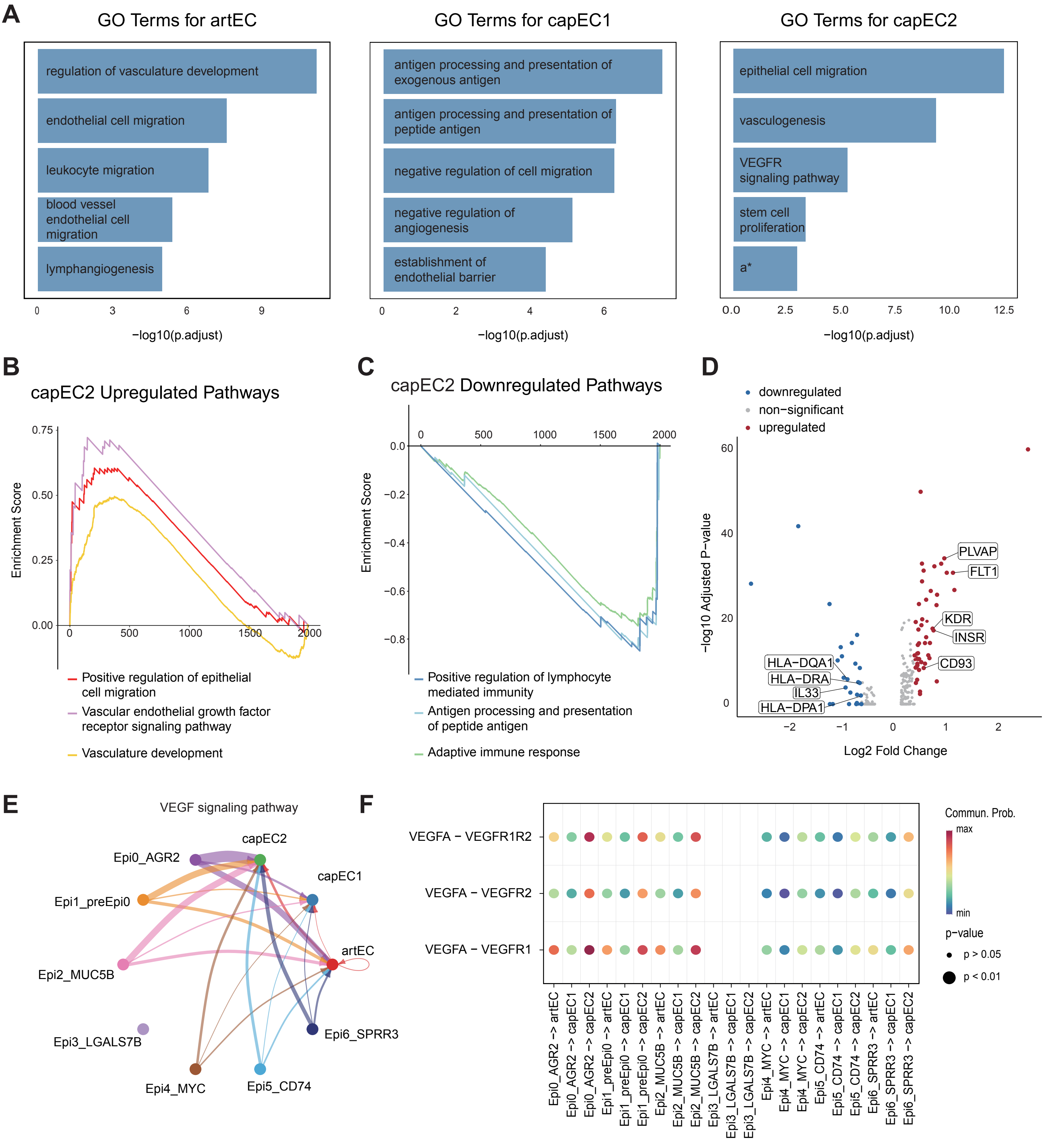
A
GO Terms for artEC
regulation of vasculature development
endothelial cell migration
leukocyte migration
blood vessel endothelial cell migration
lymphangiogenesis
−log10(p.adjust)
GO Terms for capEC1
antigen processing and presentation of exogenous antigen
antigen processing and presentation of peptide antigen
negative regulation of cell migration
negative regulation of angiogenesis
establishment of endothelial barrier
−log10(p.adjust)
GO Terms for capEC2
epithelial cell migration
vasculogenesis
VEGFR signaling pathway
stem cell proliferation
a*
−log10(p.adjust)
B
capEC2 Upregulated Pathways
Enrichment Score
Positive regulation of epithelial cell migration
Vascular endothelial growth factor receptor signaling pathway
Vasculature development
C
capEC2 Downregulated Pathways
Enrichment Score
Positive regulation of lymphocyte mediated immunity
Antigen processing and presentation of peptide antigen
Adaptive immune response
D
downregulated
non−significant
upregulated
−log10 Adjusted P−value
Log2 Fold Change
PLVAP
FLT1
KDR
INSR
CD93
HLA-DQA1
HLA-DRA
IL33
HLA-DPA1
E
VEGF signaling pathway
capEC2
capEC1
artEC
Epi0_AGR2
Epi1_preEpi0
Epi2_MUC5B
Epi3_LGALS7B
Epi4_MYC
Epi5_CD74
Epi6_SPRR3
F
VEGFA − VEGFR1R2
VEGFA − VEGFR2
VEGFA − VEGFR1
Commun. Prob.
max
min
p−value
p > 0.05
p < 0.01
Epi0_AGR2 −> artEC
Epi0_AGR2 −> capEC1
Epi0_AGR2 −> capEC2
Epi1_preEpi0 −> artEC
Epi1_preEpi0 −> capEC1
Epi1_preEpi0 −> capEC2
Epi2_MUC5B −> artEC
Epi2_MUC5B −> capEC1
Epi2_MUC5B −> capEC2
Epi3_LGALS7B −> artEC
Epi3_LGALS7B −> capEC1
Epi3_LGALS7B −> capEC2
Epi4_MYC −> artEC
Epi4_MYC −> capEC1
Epi4_MYC −> capEC2
Epi5_CD74 −> artEC
Epi5_CD74 −> capEC1
Epi5_CD74 −> capEC2
Epi6_SPRR3 −> artEC
Epi6_SPRR3 −> capEC1
Epi6_SPRR3 −> capEC2

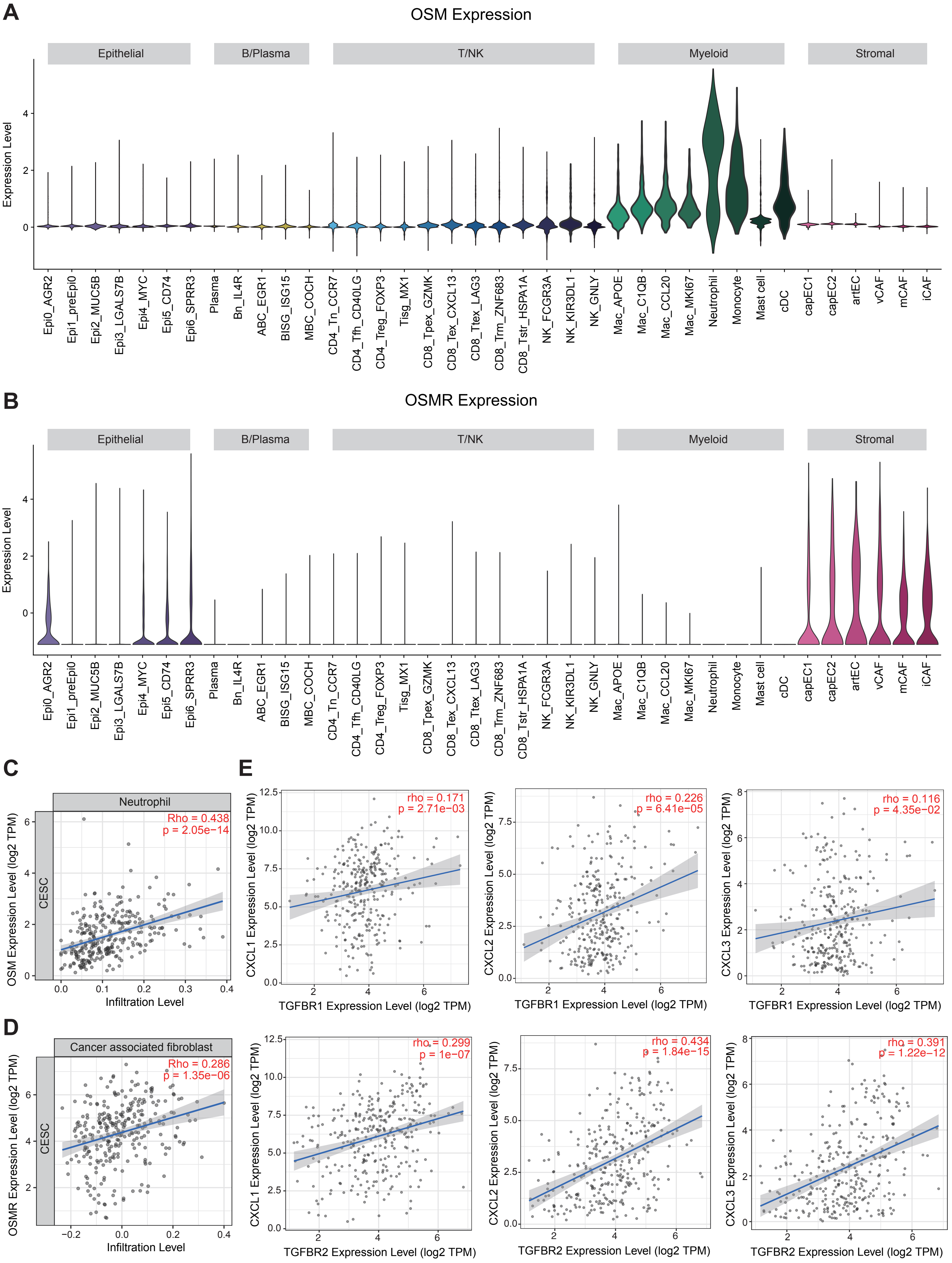

A
OSM Expression
Epithelial
B/Plasma
T/NK
Myeloid
Stromal
Expression Level
Epi0_AGR2
Epi1_preEpi0
Epi2_MUC5B
Epi3_LGALS7B
Epi4_MYC
Epi5_CD74
Epi6_SPRR3
Plasma
Bn_IL4R
ABC_EGR1
BISG_ISG15
MBC_COCH
CD4_Tn_CCR7
CD4_Tfh_CD40LG
CD4_Treg_FOXP3
Tisg_MX1
CD8_Tpex_GZMK
CD8_Tex_CXCL13
CD8_Ttex_LAG3
CD8_Trm_ZNF683
CD8_Tstr_HSPA1A
NK_FCGR3A
NK_KIR3DL1
NK_GNLY
Mac_APOE
Mac_C1QB
Mac_CCL20
Mac_MKI67
Neutrophil
Monocyte
Mast cell
cDC
capEC1
capEC2
artEC
vCAF
mCAF
iCAF
B
OSMR Expression
C
Neutrophil
CESC
Rho = 0.438
p = 2.05e−14
OSM Expression Level (log2 TPM)
Infiltration Level
D
Cancer associated fibroblast
Rho = 0.286
p = 1.35e−06
OSMR Expression Level (log2 TPM)
E
rho = 0.171
p = 2.71e−03
CXCL1 Expression Level (log2 TPM)
TGFBR1 Expression Level (log2 TPM)
rho = 0.226
p = 6.41e−05
CXCL2 Expression Level (log2 TPM)
rho = 0.116
p = 4.35e−02
CXCL3 Expression Level (log2 TPM)
rho = 0.299
p = 1e−07
TGFBR2 Expression Level (log2 TPM)
rho = 0.434
p = 1.84e−15
rho = 0.391
p = 1.22e−12

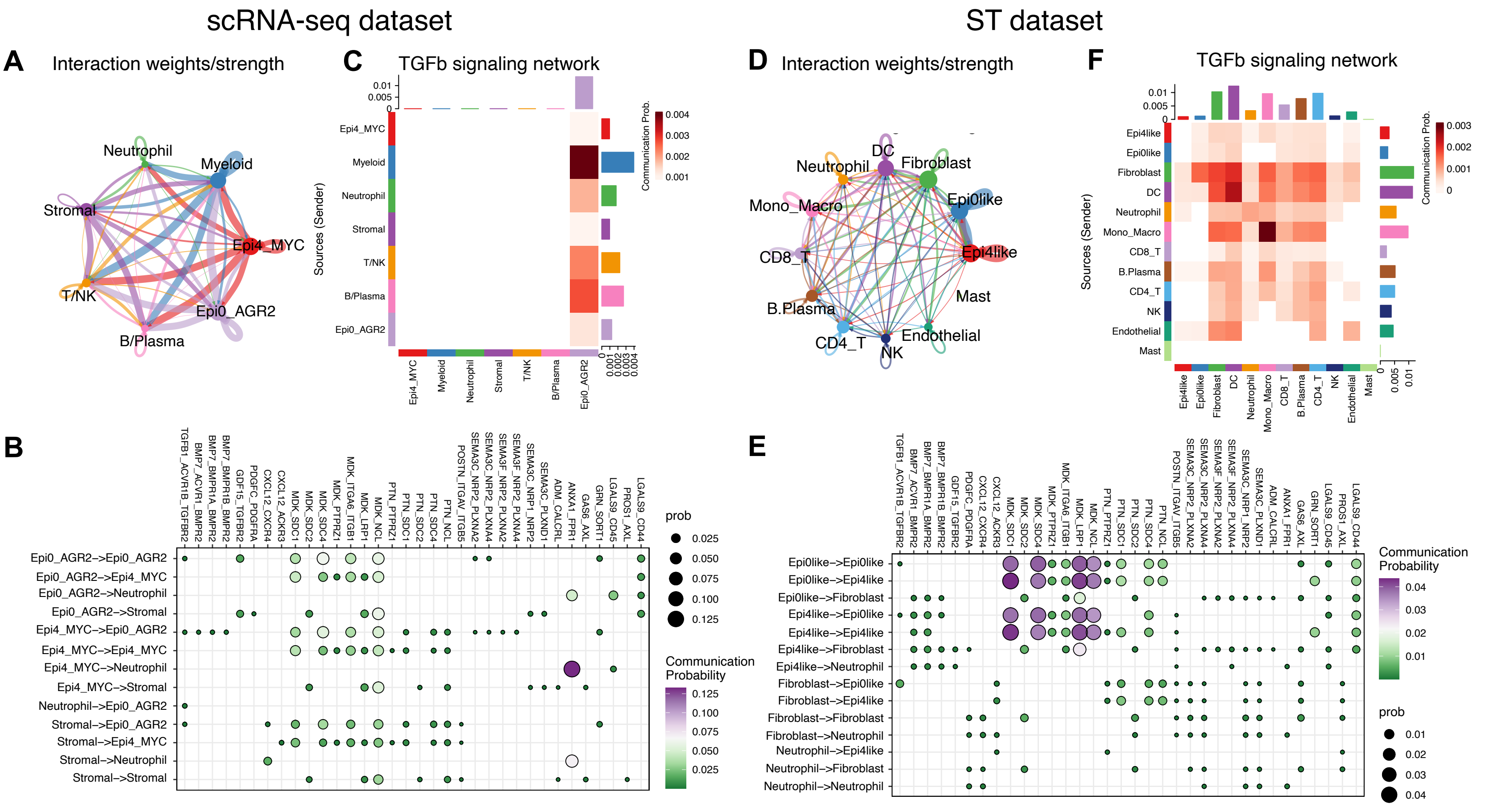
scRNA-seq dataset
ST dataset
A
Interaction weights/strength
C
TGFb signaling network
D
Interaction weights/strength
F
TGFb signaling network
B
E
Sources (Sender)
Communication Prob.
Communication Probability
prob

Supplementary Table 1. Clinical information of patients enrolled in this study

Sample	Patient	Data source	Site	Histology	Stage	Age (years)	HPV	scRNA cells
1	CC1	In house	CA	SCC	IB2	44	16, 66	743
2	CC2	In house	CA	SCC	IIIC1	39	45	3829
3	CC3	In house	CA	SCC	IB1	51	16	3944
4	CC4	In house	CA	SCC	IIA1	52	16	4502
5	CC5	In house	CA	SCC	IIIC2	32	18	4600
6	CC6	In house	CA	SCC	IB1	56	31	7888
7	CC7	In house	CA	SCC	IIB	59	16	5479
8	CC8	In house	CA	SCC	IVB	49	16	3927
9	CC9	In house	CA	AC	IIIC2	57	+	3370
10	CC10	In house	CA	SCC	IIB	51	+	5507
11	CC11	In house	CA	SCC	IIA2	77	+	9095

CA: cancer; SCC: Squamous cell carcinoma, AC: Adenocarcinoma;

Supplementary Table 2. Signature score genes for survival analysis

**Epi0_AGR2**	**Neutrophils**	**CAFs**
SERPINA1	OSM	ACTA2
AGR2	CXCR2	MCAM
CCL20	CSF3R	RGS5
CXCL2	FCGR3B	MT1A
CXCL3	IL1B	FAP
WFDC2	CXCL8	DCN
CLDN10	S100A9	CFD
MMP7	PLAUR	TAGLN
CXCL1		
KLF2		
NR4A1		

Supplementary Table 3. Information on single-cell RNA sequencing

Sample	Number of Reads	Number of Mean_Genes	Number of Cells
CC1	3,719,641	1708	743
CC2	17,909,403	1296	3829
CC3	22,305,001	1387	3944
CC4	16,152,610	1257	4502
CC5	42,074,586	2169	4600
CC6	49,664,215	1981	7888
CC7	46,023,285	2365	5479
CC8	17,900,865	1619	3927
CC9	30,196,421	2702	3370
CC10	30,778,771	1977	5507
CC11	58,039,936	2234	9095
